# Regulation of Cell Proliferation and Nrf2-Mediated Antioxidant Defense: Conservation of Keap1 Cysteines and Nrf2 Binding Site in the Context of the Evolution of KLHL Family

**DOI:** 10.3390/life13041045

**Published:** 2023-04-19

**Authors:** Gregory A. Shilovsky, Daria V. Dibrova

**Affiliations:** 1 Faculty of Biology, Lomonosov Moscow State University, 119192 Moscow, Russia; 2 Belozersky Institute of Physico-Chemical Biology, Lomonosov Moscow State University, 119234 Moscow, Russia; 3 Russian Institute for Information Transmission Problems of the Russian Academy of Sciences (Kharkevich Institute), 127051 Moscow, Russia

**Keywords:** evolution, Nrf2, Keap1, KLHL proteins, Kelch domains, longevity, bioinformatics, aging, human disease

## Abstract

Keap1 (Kelch-like ECH-associated protein 1) is one of the major negative regulators of the transcription factor Nrf2 (nuclear factor erythroid-2-related factor 2), which induces the expression of numerous proteins defending the cell against different stress conditions. Keap1 is generally negatively regulated by post-translational modification (mostly via its cysteine residues) and interaction with other proteins that compete with Nrf2 for binding. Cysteine residues in Keap1 have different effects on protein regulation, as basic residues (Lys, Arg, and His) in close proximity to them increase cysteine modification potential. In this paper, we present an evolutionary analysis of residues involved in both mechanisms of Keap1 regulation in the broader context of the KLHL protein family in vertebrates. We identified the typical domain structure of the KLHL protein family in several proteins outside of this family (namely in KBTBD proteins 2, 3, 4, 6, 7, 8, 12 and 14). We found several cysteines that are flanked by basic residues (namely, C14, C38, C151, C226, C241, C273, C288, C297, C319, and C613) and, therefore, may be considered more susceptible to regulatory modification. The Nrf2 binding site is completely conserved in Keap1 in vertebrates but is absent or located in nonaligned DA and BC loops of the Kelch domain within the KLHL family. The development of specific substrate binding regions could be an evolutionary factor of diversification in the KLHL protein family.

## 1. Introduction

Long-living species usually present more advanced and powerful systems for repairing various cellular damage, including an antioxidant protection system. Frolkis [1] called them “anti-aging systems” as they help slow down aging and increase life span. The activity of protection systems in basal conditions (as well as their ability to respond to damage) usually decreases with age [2]. The transcription factor Nrf2 (nuclear factor erythroid 2-related factor 2) is a representative of cellular antiaging agents [2,3,4]. Nrf2 regulates the transcription of antioxidant and detoxifying enzymes, which form a powerful cell defense system [2,4,5]. Nrf2 is also the main regulator of cellular homeostasis that controls the expression of more than 1% of human genes associated with biotransformation reactions (in particular, the detoxification of metabolites in the liver), redox homeostasis, energy metabolism, DNA repair, and proteostasis [4]. This factor is activated by the so-called “oxidative stressors”, as well as electrophilic agents, and provides adaptation to stress by the positive regulation of cellular antioxidant defense and other metabolic processes (for a global review, see [6]). It controls the expression of more than 200 target genes under various types of stress. The products of these genes regulate and perform many protective functions, including the detoxification and excretion of xenobiotics, pentose phosphate shunt, and autophagy [5]. Nrf2 inhibits the expression of various genes involved in inflammation by binding to their proximal regulatory regions [7]. Nrf2 (*Nfe2l2*) knockout mouse cells have increased levels of reactive oxygen species (ROS) and are more sensitive to oxidative stress [8]. In fact, Nrf2 is located at the interface between ROS signaling, endoplasmic reticulum stress response, metabolism, and autophagy. The level of Nrf2 decreases with age, and its ability to be activated in response to stress also deteriorates (for a review, see [9]). Both the level and activity of Nrf2 in the cell are not maintained at a constant level; they are subject to circadian and ultradian fluctuations (for a review, see [10]). Nrf2 activity positively correlates with the species-specific lifespan [4]. All this allows us to consider it a component of a special antiaging program; according to Lewis, it is the “guardian of healthspan and gatekeeper of longevity” [3].

One of the two main regulatory mechanisms that cause the proteasomal degradation of the Nrf2 protein is binding to Keap1 (Kelch-like ECH-associated protein 1). Previous attempts to compare sequences of Keap1 and its homologs from different organisms, to the best of our knowledge, were incomplete, affecting either only a few amino acid positions [11,12,13,14] or only two or three proteins of the Kelch-like (KLHL) family [15,16,17,18]. In this work, we aimed to describe the possible mechanisms of the antioxidant regulation of human Keap1 (primarily through the modification of its cysteine residues) from an evolutionary point of view through comparison with related proteins. We have also analyzed the conservation of the set of amino acid residues responsible for Nrf2 interaction in Keap1-related proteins. As Nrf2 inactivation contributes to the development of chronic diseases and cancer initiation, interfering with the protein binding to its negative regulator Keap1 directly or indirectly is a possible strategy for therapy [19]. We carried out a phylogenomic analysis in order to identify a complete set of Keap1 homologs that share the same domain architecture with it (the KLHL family) and are encoded in vertebrate genomes. In the course of our analysis, we found several groups of enzymes that were previously erroneously assigned to another family and analyzed the conservation of several key residues in different groups of Keap1 homologs on the phylogenetic tree.

### 1.1. Nrf2 Regulators

**Figure 1:**
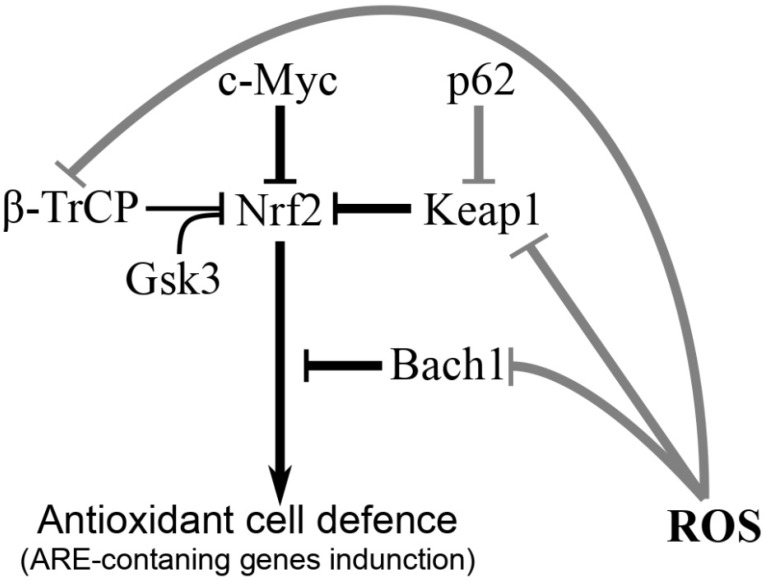
Regulation of Nrf2 transcription factor that controls expression of more than 200 cytoprotective enzymes responsible for detoxification and antioxidant defense. Figure adapted from [9]. Nrf2 activation is caused by reactive oxygen species (ROS). Glycogen synthase kinase 3 (Gsk3), activated via multiple signaling cascades, acts as a suppressor by inhibiting Nrf2. Grey lines with blunt ends —Nrf2 stimulation (and subsequent expression of antioxidant enzymes) through inhibition of its inhibitors; black lines with blunt ends—Nrf2 inhibition; arrow—direct stimulation. Designations: ROS, reactive oxygen species; Bach1, BTB domain and CNC homolog 1; Gsk3, glycogen synthase kinase 3; Keap1, Kelch-like ECH-associated protein 1; Nrf2, nuclear factor erythroid 2-related factor 2; β-TrCP, β-transducin repeat-containing protein.

Nrf2 is regulated at the protein level by several ubiquitin ligases [20] and post-translational modification [21,22,23,24,25] (Figure 1). 

Both main negative regulators of Nrf2, namely the ubiquitin ligase adapter Keap1 [20,26] and Gsk3/β-TrCP [27], promote the ubiquitination of Nrf2, and while the nature of its action, the mechanism of double phosphorylation, and more than 100 substrates are known for Gsk3 (for a review, see [5]), much less is known about the function and substrates of Keap1 [28]. 

Nrf2 is also inhibited by the c-Myc protein [29]. The Bach1 protein does not directly inhibit Nrf2 but rather competes with it for binding to the ARE (antioxidant response element) sequence [28] (Figure 1). Nrf2 could be phosphorylated by various protein kinases, which either causes its inactivation and export from the nucleus (Fyn kinase) or, vice versa, promotes its translocation to the nucleus (casein kinase 2 and protein kinase C) [30,31]. Under nonoxidative stress (for example, caused by the tetrafluoroethyl cysteine), Nrf2 activation can be achieved by phosphorylation through ER-mediated protein kinases, such as PKR-like endoplasmic reticular kinase (PERK) [32].

### 1.2. Keap1 Is a Negative Regulator of Nrf2

**Figure 2:**
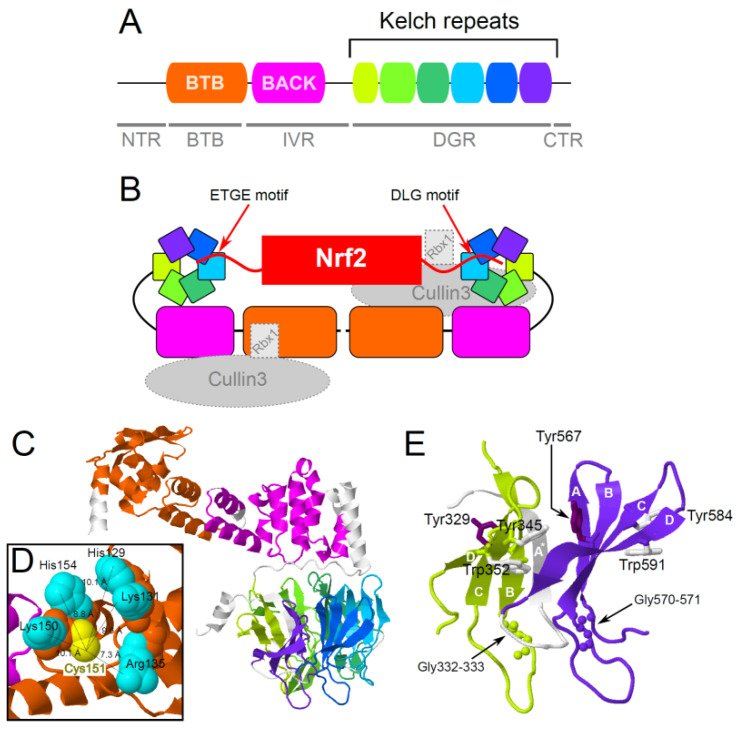
**Keap1 domain structure**. (**A**) Human Keap1 protein with positions of conserved domains according to Pfam search and literature data. Positions of BTB (PF00651), BACK (PF07707), and 6 Kelch domains (PF01344) are shown according to the boundaries of hits of the corresponding Pfam domains. (**B**) Scheme of binding between the Keap1 dimer, its target protein Nrf2, and other components of ubiquitin ligase: Cullin 3 and Rbx1 (based on [15]). (**C**) Prediction of the spatial structure of the human Keap1 protein by AlphaFold [39]. Pfam domain hits are mapped onto the structure. Poorly predicted N-terminal region is not shown. (**D**) Base residues H129, K131, R135, K150, and H154 are thought to be important determinants of the “reactivity” of C151 in terms of oxidative stress. (**E**) The first (shown in lime) and the last (shown in purple) of the Kelch domains with highly conserved residues. Notice how the β-strand following the last Kelch domain is inserted into the β-sheet of the first Kelch domain. The following residues are depicted for each Kelch domain: (1) tyrosine residue from the conserved YxxGG motif located in the second β-strand (βB) is shown by dark purple sticks, and glycine residues on the tip of it are depicted with balls; (2) conserved Tyr residue, which provides stacking for the super conserved Trp residue belongs to the third β-strand (βC) and is shown by white sticks; (3) Trp residue is shown by white sticks and belongs to the fourth β-strand (βD). Spatial protein structures were visualized with Jmol viewer (http://www.jmol.org/).

Keap1 is widely distributed in eukaryotes and, at least in vertebrates, has the same function. It is expressed in a wide variety of cell types and tissues [33]. It is mainly located in the perinuclear region of the cytoplasm and in small amounts in the nucleus and endoplasmic reticulum [34]. Its amount varies from 50,000 to 300,000 molecules per cell, while the level of Nrf2 is 2–3 times lower [35]. Keap1 assembles as a homodimer through its BTB domains into a stable and functional E3 ubiquitin ligase complex with Cul3 and Rbx1 proteins (Figure 2A,B). The lysine residues intended for polyubiquitination in Nrf2 are in close proximity to the interacting domains of Keap1. Thus, Keap1 negatively controls Nrf2 activation by promoting Nrf2 degradation through the Cul3-Rbx1-Keap1 ubiquitin ligase complex, thereby preventing Nrf2 activity under stress-free conditions [20,26]. Keap1^−/−^ mice show postnatal lethality (death at the weaning stage) due to Nrf2 hyperactivation [36].

#### 1.2.1. Keap1 Structure

Keap1, according to the literature, consists of five conserved domains (Figure 2A,C): (1) the N-terminal region (NTR); (2) the BTB/POZ domain (broad complex, Tramtrack, Bric-a-brac/Pox virus, and zinc finger domain) responsible for the dimerization and assembly of the Cul3-Rbx1-Keap1 ubiquitin ligase complex [37]; (3) an intermediate IVR domain (intervening region), including the BACK domain and linker regions, which contains oxidation-sensitive cysteine residues and the NES (nuclear export signal) motif (residues 301–310 in human Keap1) [38]; (4) DGR (double glycine repeat) domain consisting of six repeats, which form the structure of a six-bladed β-propeller responsible for Nrf2 binding; and (5) the C-terminal region (CTR). Further interaction with Cul3 is provided by the so-called 3-box motif (residues 181–212 in human Keap1), which is located at the beginning of the IVR domain [15].

#### 1.2.2. Keap1 Binds Nrf2 and Prepares it for Ubiquitination

In the absence of pronounced oxidative/toxic stress conditions, Keap1 is the main cytosolic regulator of the Nrf2 protein. Keap1 regulation of Nrf2 also occurs in the nucleus after induction, promoting its nuclear export by effectively turning off Nrf2-dependent transcription. Keap1 is able to recognize and bind Nrf2, and this results in the lysine residues in Nrf2 being prepared for the covalent attachment of ubiquitin. Keap1 interacts with two specific amino acid sequences in Nrf2, docking it for ubiquitination via the Cul3-Rbx1-Keap1 ligase complex. The DGR domain of Keap1 interacts with the N-terminal Neh2 domain of Nrf2, namely with two amino acid regions, in a hinge-and-latch mechanism. These two motifs, ETGE and DLG, have different binding affinities for Keap1. ETGE shows a slow rate of association and dissociation with high binding affinity compared to the DLG sequence, which binds faster but with about a 100-fold lower binding affinity. High-affinity ETGE is hypothesized to act as a hinge, anchoring Nrf2 to the Kelch domain, while DLG acts as a latch. Keap1 modifications in response to oxidative stress may prevent latch attachment, reducing the regulatory effects that Keap1 has on Nrf2 [40]. In human Keap1, the following residues are responsible for the Nrf2 binding: for the ETGE motif, the first Glu (Glu79) interacts with Keap1 residues Arg415, Arg483, and Ser508, while the second Glu residue (Glu82) interacts with Ser363, Asn382, and Arg380; the DLG motif binds via aspartate to the same Arg415, while Asn414 can be involved in the binding of cognate residues [41].

#### 1.2.3. Mechanisms of Regulation of Keap1

Post-translational modifications, such as oxidation, phosphorylation, S-nitrosylation, ubiquitination, alkylation, carbonylation, glycosylation, S-glutathionylation, succination, and sulfhydrylation functionally inactivate Keap1 and, thus, serve as an alternative way to activate Nrf2, resulting in the increased transcription of Nrf2 target genes [21,22,23,24,25]. Most known Nrf2 inducers are electrophiles that covalently modify cysteine residues present in the thiol-rich domain of Keap1 [42] via oxidation or alkylation. Thus, Nrf2 can be physiologically activated by an increase in oxidative stress, but the same effect can be caused exogenously by chemical agents [43].

A distinctive feature of the Nrf2/Keap1 system is a large number of cysteine residues capable of oxidative modification, which respond quite specifically to various stimuli. Cysteines are unique amino acids because of their sulfhydryl (thiol) functional group that performs various functions, including (1) forming intra- and intermolecular covalent bonds with other Cys thiols, (2) binding to metals and metalloids, and (3) reversible or irreversible oxidation upon reaction with oxidants [44]. Under conditions of oxidative stress, Keap1 repression of the Nrf2 protein is blocked. As a result of the modification and inactivation of Keap1, the cellular level of Nrf2 increases many times [40]. This allows Nrf2 to move into the nucleus and accumulate there to activate the transcription of antioxidant proteins and deal with increased levels of ROS. The attenuation of Nrf2 suppression by Keap1 depends on the ability of Keap1 to act as a redox sensor.

Some modifications inactivate Keap1 but do not destroy the complex with Nrf2, and, therefore, a large number of Nrf2 molecules remain bound to Keap1 in an inactive state. In contrast, the interaction of the Keap1 cysteine thiol groups with heavy metals causes not only the stabilization of Nrf2 but also its release from the complex with Keap1 [45,46].

The processes of the oxidative/electrophilic modification of thiol groups of several cysteine residues in Keap1 play a key role in the removal of repression from Nrf2 [47]. These residues include cysteines at positions 226, 434, and 613 and especially C151, C273, and C288 (hereinafter, numbering is provided for the human enzyme) [48]. Each of them responds to various oxidizing agents, leading to the concept of the “cysteine code” (the hypothesis that different inducers modify certain combinations of cysteines in order to tightly control the Keap1/Nrf2 stress-sensitive response [49]). For example, four cysteine residues (C226, C613, C622, and C624) are important in the response to hydrogen peroxide. Analysis of mouse embryonic fibroblasts expressing a series of Keap1 mutants in these residues showed that Keap1 uses these cysteine residues in excess to implement a fail-safe mechanism that allows any combination of C226, C613, and C622/C624 to form a disulfide bond for detecting hydrogen peroxide [14].

The main determinants of the high reactivity of C151 are the basic amino acids, H129 K131, R135, K150, and H154, localized in spatial proximity to it, which seem to contribute to the stabilization of the residue in the form of a thiolate anion (Figure 2D) [21,48]. 

Keap1 itself also undergoes ubiquitination, which predisposes it to proteasome-independent degradation, thus promoting Nrf2 activation [50]. The prolonged exposure of Keap1 to oxidative stress is also believed to lead to further modifications of Keap1 that expose lysine residues for its own ubiquitination [51] and p62-dependent degradation [52]. 

#### 1.2.4. Nrf2 Inductors Acting through the Post-Translational Modification of Keap1

Keap1 inhibitors (and, thus, Nrf2 inducers) are divided into five classes based on their reactivity profile towards the three most reactive Keap1 cysteines (C151, C273, and C288) [12,53,54,55]. Residues C151 and C273 are the most reactive cysteine residues in Keap1 and undergo various modifications that strongly affect Keap1 activity [21,24,25,45,46,56,57,58,59,60,61,62,63,64]. For instance, C151 and C273 residues undergo phosphorylation [59] and, together with C434 [62], interact with (bio)NO donors, being “nitric oxide sensors” [21,24,56,57,58]. Residues C151, C257, C273, C288, and C297 are modified by alkylating agents, including the mitochondrial metabolite itaconate [25]. C151 and R135 are capable of forming a methylimidazole crosslink in the presence of methylglyoxal, which is typical for diabetes [61]. Residues C226 and C613 form disulfide bridges in the presence of metals (As^3+^, Cd^2+^, Se^4+^, and Zn^2+^) and hydrogen peroxide, as well as in the presence of H_2_S [21,45,46]. C77, C434 and, to a lesser extent, C23, C38, C226, and C273 covalently bind PBA (pubescenoside A, 2-(trans-caffeoyloxy)methyl-3-hydroxy-1-butene-4-O-beta-D-glucopyranoside) with their thiol group [63]. Interestingly, the Keap1 C23Y mutation abolishes its ability to repress Nrf2 [64]. 

However, cysteines in Keap1 are not the only residues prone to modification. For instance, the conserved serine S104 undergoes S-glutathionylation [60], while K131 undergoes malonylation, acetylation, and palmitoylation [58]. 

In addition to somatic mutations in Keap1 genes, several other mechanisms have been found to cause Nrf2 stabilization [65]. For example, the inactivation of fumarate hydratase in renal carcinomas leads to Nrf2 activation through succination of Keap1 cysteine residues by accumulated fumarate [66].

### 1.3. KLHL Protein Family and Its Mutations as an Oncogenic Factor

Keap1 belongs to the BTB-Kelch family of proteins, which includes about fifty members divided into two subfamilies: Kelch-like 1–42 (KLHL1–42) and Kelch containing the BTB domain 1–14 (KBTBD1–14) [15,67]. These proteins differ in domain architecture: members of the KLHL family must also have the BACK domain, while those of the KBTBD family lack it [68].

All of these proteins assemble with Cullin 3 (Cul3) and RING-box protein 1 (Rbx1) to form Cullin–RING multisubunit ubiquitin ligases; their BTB domains are responsible for dimerization and interaction with the Cul3-E3 ligase [67,69]. Thus, KLHL family proteins typically contain the BTB and BACK domains, as well as 5–6 Kelch motifs combined into the so-called DGR region (Figure 2A,C). KLHLs tend to target tissue-specific proteins for degradation, thereby affecting differentiation, homeostasis, metabolism, cell signaling, and response to oxidative stress. 

The Kelch motif is an ancient sequence motif also present in prokaryotes and widespread in eukaryotes, about 50 amino acids long. Often it can be encountered as five to seven repeats that form a β-propeller tertiary structure [15]. Protein K13 with a BTB domain and a DGR domain with six Kelch repeats exists already in *Plasmodium* [70]. 

Mutations in some KLHL genes are harmful and often lead either to the formation of genetic diseases or cancer in humans. Keap1 (KLHL19) and three other members of the KLHL family are linked to tumor genesis: KLHL6, KLHL20, and KLHL37 (ENC1). Numerous Keap1 mutations are associated with poor prognosis, as they promote tumor cell proliferation and cause resistance to chemotherapy in various types of cancer (see [71,72] for a review). A decrease in Keap1 expression unlocks Nrf2 transcriptional activity, which increases the expression of proteins that contribute to the survival and proliferation of cancer cells [73,74].

Numerous mutations (such as insertions and nonsynonymous substitutions) characterize the coding region of the Keap1 gene in cancer cells and have been identified in liver, gallbladder, and lung cancer cells [75,76,77,78,79]. Interestingly, these mutations are evenly distributed throughout the gene and rarely cause protein truncation. Instead, they lead to a change in the ability to bind and regulate Nrf2 degradation. 

Nuclear matrix protein KLHL37 (nuclear-restricted protein/brain (NRP/B); ectodermal-neural-cortex 1 (ENC1)) is an actin-binding protein expressed predominantly in nerve cells [80] and the brain [81]. 

Studies have confirmed that ENC1 is activated in various types of brain cancer, as well as in colon, ovarian, and breast cancers. However, it has been shown that ENC1 significantly increases the level of ROS in ovarian cancer cells, inhibiting the proliferation, invasion, and migration of these cells [82]. ENC1 binds to Nrf2 and can increase ARE-dependent gene expression [83,84]: Nrf2 accumulates in the nucleus, and its export to the cytoplasm, as well as Keap1-dependent degradation, is prevented. However, ENC1 can also reduce the expression of ARE-dependent genes [85] through p62 binding and disruption of Keap1 degradation [80]. Mutations of the ENC1 protein, which change its localization from nuclear to cytoplasmic, disrupt the induced expression of ARE-dependent genes. Mutations described in ENC1 are mainly located in the Kelch domain but also exist in the BTB and BACK domains [81] and are associated with brain tumors [86].

## 2. Materials and Methods

### 2.1. Phylogenomic Analysis of the KLHL Family in Vertebrates

A characteristic feature of the KLHL family is the presence of two conserved domains, namely BTB and BACK, and a region with several Kelch repeats. The latter exists in many other proteins and may produce nonspecific hits in sequence searches, thus we decided to use a combination of the two former domains for the identification of KLHL family proteins.

We used the **Jalview** [87] program for all manipulations with multiple alignments as well as our own software (https://github.com/udavdasha/alnalyser, (accessed on 14 April 2023)).

#### 2.1.1. Construction of the HMM Profile for the N-Terminal Region of KLHL Proteins

We took all known human proteins related to the KLHL family (42 proteins in total), aligned them with **Muscle [88]**, and constructed an profile HMM with **HMMer 3.1b1 [89]** for a low-selectivity search of all related proteins. 

We applied this profile to search in a short representative sample of vertebrate proteomes, namely taken from *Danio rerio* (fish), *Xenopus tropicalis* (frog), *Gallus gallus* (bird), *Alligator sinensis* (reptilian), *Homo sapiens* (human), *Mus musculus* (mouse), *Bos taurus* (cattle), *Balaenoptera musculus* (blue whale), and *Heterocephalus glaber* (naked mole-rat). We obtained 551 proteins, removed those that did not contain BTB and BACK domains, and aligned the remaining 466 sequences. We then selected the N-terminal region encompassing BTB and BACK domains and constructed an HMM profile for a more selective search.

#### 2.1.2. Hit Search with Profile Covering BTB and BACK Domains in Vertebrates

First, we identified all possible hits of the profile in the aforementioned vertebrates using a loose e-value threshold of 10^−5^: a total of 1246 sequences after short isoform filtration. Then, we selected only those hits which suited the KLHL family by their domain architecture. For this, we used the **Domain Analyser** service (https://depo.msu.ru/module/domainanalyser, (accessed on 14 April 2023)) and the **Pfam** profile database [90]. All proteins that contained BTB, BACK, and at least one Kelch repeat domain at a very loose e-value threshold of 0.1 were sampled, yielding 449 proteins. We did not expect to find all six Kelch repeats in the proteins of interest through domain search. Several human proteins known to belong to the KLHL family contain hits of any Pfam domain for only some of these Kelch repeats (e.g., only four or three of the six Kelch domains are detected in KLHL34 or KLHL11, respectively). Therefore, we cannot rely on the presence of hits of all Kelch domains as a characteristic trait of the KLHL family. To check if our search was sensitive enough, we examined the **Ensembl** database [91] for human KLHL members (42 proteins) and checked that all of these proteins had fallen into our sample. Then, we aligned all proteins using Muscle once more. 

#### 2.1.3. Phylogenetic Tree Construction

For the phylogenetic tree construction, we used the **MEGA7** program [92]. The tree was constructed according to the neighbor-joining algorithm, and the JTT method was used to estimate evolutionary distances and uniform rates among sites. We used manually selected regions (conserved blocks) consisting of 202 positions for tree construction and conducted a bootstrap test for the tree branches. 

### 2.2. Statistical Analysis of Multiple Alignments

We studied human KLHL proteins and counted the most frequently occurring amino acids in each position in multiple alignments. In multiple alignments of the human KLHL proteins, we have ranked all positions according to their maximal frequency of co-occurrence of the most common amino acid residue. 

To minimize the noise, positions that ranked less than 33% were excluded from further consideration. Positions with a rank of 66% or higher were considered ultraconservative, those with a rank between 50 and 66% conservative, while those with a rank between 33 and 50% weakly conserved. 

Next, we compared all sequences with the consensus that had the most frequently occurring amino acid in each position. In addition, we compared it with a reference protein (in this case, Keap1 (KLHL19)). For the mismatch of amino acids with the consensus or reference protein, we charged penalties (which means that these proteins begin to diverge through evolution). The penalty is greater the more often lost/substituted amino acids occur (i.e., proportional to frequency). This makes it possible to distinguish a situation when a protein lost a few poorly conserved fragments from a situation when it lost specific functionally and structurally important amino acids. We searched for positions with a high frequency of occurrence of two amino acids at once, for example, in neighboring clades (that is, the replacement was not random but could represent protein specification in evolution). Comparisons were made for Kelch domains, cysteines, and some other positions important for functional activity; in total, about 275 positions were selected.

## 3. Results

### 3.1. Phylogenetic Analysis of the KLHL Protein Family Suggests New Possible Members

**Figure 3:**
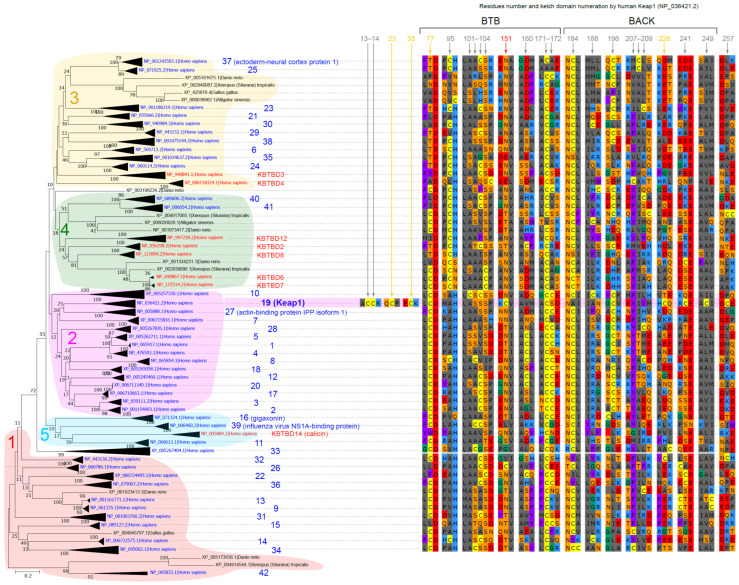

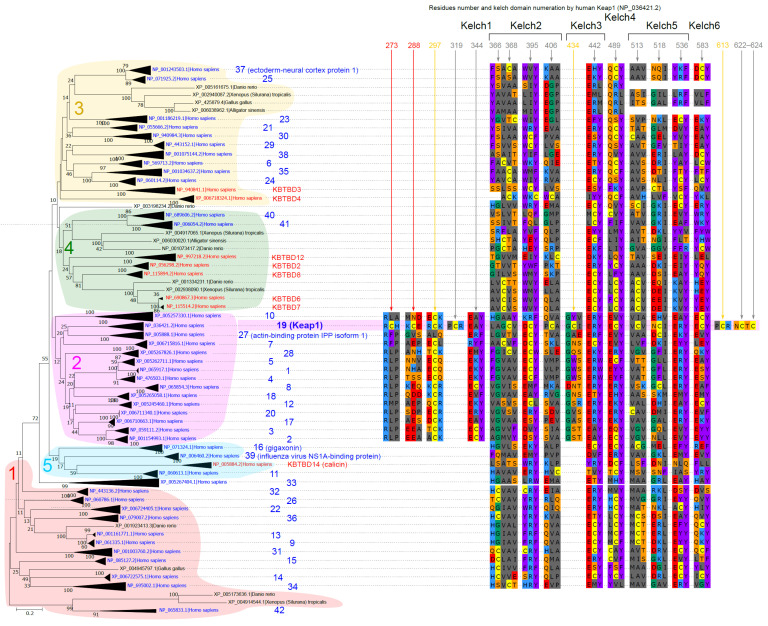
**Short version of the phylogenetic tree of KLHL proteins with cysteines and their neighboring residues** (see Figure S2 for the full version). Clades containing human proteins are collapsed into a single sequence. All human Keap1 cysteine residues and their flanking neighbors are mapped onto the tree, as well as in other KLHL proteins.

The phylogenetic tree of the KLHL family (Figure 3) can be classified into four main clades (which are consistent with previous works [67,69]) and one lesser clade. According to our data, several proteins, which were previously attributed to basal positions inside these four main clades, formed a separate fifth clade.

To our surprise, several proteins that were previously annotated as members of the KBTBD subfamily possessed all the necessary features required to enter the KLHL subfamily, namely they contained both BTB and BACK domains as well as several Kelch domains. These proteins did not cluster out from acknowledged members of the KLHL subfamily on the tree but rather clustered within the typical KLHL clades. We suggest that they should be considered typical members of the KLHL subfamily; however, in this paper, we adhered to their original annotation to avoid confusion. 

During our investigation, we encountered a peculiar human protein (NP_006758.2 or LZTR1_HUMAN), which technically contains all domains required to be called a member of KLHL, specifically BTB, BACK, and Kelch, but it is circularly permutated; thus, it contains Kelch repeats on the N-terminus instead of the C-terminus. This protein mediates Ras GTPase ubiquitination [93], which is more or less the typical function of other members of the KLHL family. In multicellular eukaryotes, the region with the BTB and BACK domain in this protein is duplicated, while proteins from different unicellular species contain a single BTB-BACK region (Figure S1). At the sequence level, this protein appears to diverge from other members of the KLHL family but at the same level as the KLHL42 protein.

For all proteins annotated as belonging to the KBTBD family, we observed typical BACK domains (Figure S2), with all important residues generally conserved (Figure S3). It is worth mentioning, however, that the KBTBD2, KBTBD8, KBTBD6, and KBTBD7 conserved motifs in the Kelch repeats are modified: they typically do not contain a double glycine repeat in the first and the last piece of the β-propeller. Similar discrepancies, however, are observed in KLHL members, e.g., KLHL40 and KLHL41. KBTBD6 and KBTBD7 raise the most questions as these proteins lack double glycine residues in almost all of their blades (Figure S3), and only a single Kelch domain can be predicted in them with the Pfam database (Figure S2). However, AlphaFold still confidently predicts that they have six blades in their DGR domain, but a further comparison of their structures is required to confirm multiple alignments. Thus, our analysis suggests that these proteins should be considered normal members of the KLHL family, into which they fit both evolutionary and formally.

Clade 1 contains KLHL9, KLHL13, KLHL14, KLHL15, KLHL22, KLHL26, KLHL31, KLHL32, KLHL34, KLHL36, and KLHL42. This clade is listed as a part of Clade 3 by Ehrlich et al. [69]. Other proteins of this clade include KLHL33, KBTDB11, and KBTBD13, although KBTDB11 and KBTBD13 lack the BACK domain and, thus, were not included in our analysis, whereas KLHL33 cannot be considered part of any clade. We also noticed that KLHL42 differs greatly from the other members of the KLHL family and has multiple truncated motifs in both the dimerization BTB domain and DGR domain. For the human KLHL42 protein, the BTB domain hit has a very poor e-value of about 0.05 compared to, for example, 10^−30^ for Keap1.

Clade 2 contains KLHL1, KLHL2, KLHL3, KLHL4, KLHL5, KLHL7, KLHL8, KLHL10, KLHL12, KLHL17, KLHL18, KLHL19 (Keap1), KLHL20, KLHL27, and KLHL28. The only difference with the data from Ehrlich et al. [69] is the position of KLHL16 (gigaxonin) which, according to our analysis, belongs to separate Clade 5.

Clade 3 contains KLHL6, KLHL21, KLHL23, KLHL24, KLHL25, KLHL29, KLHL30, KLHL35, KLHL37, and KLHL38 and is thus far fully compatible with the previously published tree (Clade 4 in Ehrlich et al. [69]); our data suggest that the proteins KDTDB4 and KDTDB3 can also be added to this clade.

Clade 4 contains KLHL40, KLHL41, KBTDB2, KBTDB6, KBTDB7, KBTDB8, and KBTDB12. It is almost the same as Clade 1 denoted by Ehrlich et al. except for KDTDB4 and KLHL11 proteins. KDTDB4 on our tree rather clustered into Clade 3. KLHL11 also does not belong to this clade, and the tree topology of Ehrlich et al. [69] actually does not group it to Clade 1; it is rather considered a member of this clade erroneously. According to our analysis, KLHL11 falls into separate Clade 5.

Clade 5 contains KLHL11, KLHL16, KLHL39, and calicin. While calicin was missing from previous trees, the three latter proteins occupied basal places in Clades 2 and 4.

### 3.2. Analysis of Conservation of Cysteine Residue Conservation in KLHL Proteins: The “Cysteine Code”

We analyzed the conservation of cysteine residues in Keap1 (see the discussion in Section 4.1 for the reasoning behind the residue conservation analysis in Keap1 and the whole KLHL family simultaneously). The results of this analysis are summarized in Figure 3 and Table 1. Keap1 shares several conserved Cys residues either with the entire KLHL family (namely, C77, C171, and C196) or just with the Clade 2 members (C297). Several conserved cysteine residues are missing from Keap1 (namely, cysteine in positions 172 and 184 and several variably positioned conserved cysteines inside regions 101–104 and 206–208). 

However, the vast majority of cysteines known to play a role in the inactivation of Keap1 by oxidative stress (see Section 1.2.4) are highly conserved, with a slight possible variation between the Keap1 proteins of different species only. This is consistent with the suggestion that Keap1 likely developed its own “cysteine code” for reacting on specific oxidative conditions, which should trigger its deactivation (and, thus, the activation of the protective Nrf2) but not the deactivation of other members of the KLHL family.

While analyzing our data, we found a certain discrepancy between the domain boundaries provided by the Pfam profile HMM search and those presented on the UniProt page of human Keap1. Namely, the coordinates of the BTB domains differed substantially (with the BTB predicted by the Pfam profile having coordinates 67–178 and the UniProt BTB having coordinates 77–149), and the coordinates of Kelch domains shifted by around 10 residues (for instance, Kelch6 had the coordinates 553–597 in our Pfam search and 565–611 in the UniProt record). To choose the correct version, we mapped the corresponding coordinates onto the full-length structure of the protein that was predicted using AlphaFold [39]. The results are presented in Figure S6 in Supplementary File S1. According to these results, the Pfam domain prediction appeared to correspond better with the structural data. First, the BTB domain in the Pfam prediction covers all spatially separated N-terminal parts of the protein, whereas the UniProt prediction contains a loop between the secondary structure elements but stops arbitrarily just before the conserved α-helix (Figure S6A). Second, the UniProt prediction for the components of the β-propeller splits them so that the first β-strand A of each β-sheet belongs to the previous Kelch domain (Figure S6B).

Interestingly, the DGR domain of Keap1 does not appear to contain strong cysteines at all.

### 3.3. Conservation Analysis in the DGR Domain, Nrf2 Binding Sites, and BTB Domain

As we mentioned before, all known residues within the Kelch repeats (i.e., blades of the β-propeller), which are responsible for the structural packing of the domain, are very well-conserved inside the KLHL family (Figure S3). 

#### 3.3.1. Structural Elements of the DGR Domain and Their Conservation at the Sequence Level

First, it is the YxxGG motif, whose double glycine residues are located at the beginning of the loop between the βB and βC strands (BC-loop), while the conserved tyrosine faces the next blade (Figure 2E). In four out of the six inter-blade regions of human Keap1, it forms a hydrogen bond with the Asp or Asn residue located five residues after itself in the loop between βC and βD [94]. Second, there is a conserved tyrosine residue, which is located on the end of the βC strand. Perhaps it interacts with the tyrosine from the YxxGG motif from the previous Kelch domain; however, the distance between their OH groups (around 5Å) and the angle between them does not encourage direct hydrogen bonding. They may still bind each other through a water molecule, and, indeed, we may see bridging water molecules between these residues [94]. This Tyr residue is also involved in the stacking interaction with the last residue of the Kelch motif: the tryptophan at the beginning of the βD strand. 

This Trp is almost absolutely conserved in the KLHL family. It forms a hydrogen bond with the DA loop of the previous blade either via the OH group of Thr residue or the oxygen of the main chain. The detailed statistics of the occurrence of this Trp residue in human KLHL proteins can be found in Supplementary File S2. Several other interactions also contribute to the structural stability of the DGR domain.

However, despite the crucial role of the aforementioned residues and their overall conservation, they can still be substituted. The first Kelch domain is particularly poorly aligned between various KLHL proteins and frequently misses the tyrosine from the YxxGG motif (less than 20% of the human KLHL proteins preserve it). However, this Tyr residue is present in Keap1, KLHL1, KLHL4, KLHL18, and KLHL27. 

This residue can also be replaced by phenylalanine. The Y375F substitution in the second Kelch domain is typical for proteins of Clade 1. The Y473F substitution in the fourth Kelch domain occurs in about half of Clade 3, and Y567F (the sixth Kelch domain) and even Y567H are found in the proteins of Clade 3. Even the double glycine residues which gave their name to the DGR domain can be missing in certain cases in different Kelch domains (e.g., several Clade 3 and Clade 4 proteins miss this motif in the sixth Kelch; in the KLHL11 protein, this motif is missed simultaneously in Kelch domains 2, 4, and 5). The first structures of the Kelch domains have shown that glycine residues in this motif have backbone angles that could be easily achieved by glycine only, while other residues would occupy unfavorable angles on the Ramachandran plot [94]. However, if only a single site has a disturbance in the region, perhaps the protein can still fold correctly. The detailed statistics of the occurrence of glycine within the GG repeats following the βB strand in human KLHL proteins can be found in Supplementary File S2.

#### 3.3.2. Conservation of the Binding Site between Keap1 and Nrf2

Specific binding between the amino acid residues of Keap1 and Nrf2 (which was briefly described in Section 1.2.2) is accompanied by several contacts between the protein backbones, which are less sensitive to the amino acid substitutions. 

A known feature of the Kelch repeats is their high variability in certain regions, which form loops between the β-strands B and C (BC loop) and the β-strands D and A (DA loop, where A comes from the next Kelch repeat). The loops do not only differ in sequence but even more in length; thus, these regions usually cannot be aligned between different members of the Kelch family. The only exception we have found is the region between the second and the third Kelch repeat. Some regions, however, can be aligned on the level of Clade 2, to which Keap1 belongs. We analyzed the conservation of specific Nrf2 binding residues on the levels of Keap1, Clade2, and all KLHLs (Table 2, Figure S5) and found that only one of them, namely Ser508, is partially conserved in Clade 2, whereas all other residues are not conserved between various KLHL proteins. Indeed, they are completely conserved within the Keap1 clade. 

Thus, as expected, catalytic amino acids responsible for binding to target proteins are extremely different in various members of the KLHL family, which further emphasizes the specificity of these ubiquitin ligase adapters. The same was previously observed for positions associated with the so-called “superbinding” hypomorphic mutations of Keap1 “ANCHOR” (named after Additionally NRF2-Complexed HypomORph, these are R320Q, R470C, G423V, D422N, G186R, S243C, and V155F). These mutations promote the interaction of Keap1 and Nrf2, most likely due to a decrease in flexibility at the contact points between the proteins [70,95].

Proteins containing the KRR motif (mimicking the Keap1 arginine triad R380, R415, and R483), such as the inhibitor of cyclin-dependent kinase 1A (CDKN1A or p21) [96], have also been shown to compete with Keap1 for binding to Nrf2. Interestingly, the KRR sequence motif could be observed in several KLHL proteins, predominantly from Clade 2, but its location is not conserved. Several proteins have this motif in the same position corresponding to the 505–507 residues of Keap1, namely KLHL1 (residues 635–637, hereinafter in numeration of the human protein), KLHL4 (residues 605–607), and KLHL5 (residues 677–679). Two other proteins share this motif in a different position corresponding to the 361–363 residues of Keap1, namely KLHL7 (residues 338–340) and KLHL12 (residues 356–358), while in KLHL20, the KRR motif (residues 428–430) is aligned with residues 360–362 of Keap1. The KLHL8 motif (residues 402–404) corresponds to Keap1 412–414, while the KLHL27 motif (residues 214–216) corresponds to Keap1 259–261 residues.

The presence of these conserved KRR motifs in different members of the KLHL family could point to their possible interaction with Nrf2; thus, they could compete with Keap1 for Nrf2 binding and interfere with Keap1-dependent Nrf2 regulation. Although this is possible, such a mechanism is unlikely to be evolutionarily old, as KRR motifs are located in different places in the protein sequences of KLHL proteins.

#### 3.3.3. Conservative Residues in the BTB Dimerization Domain

The conservation of several other functionally important residues can be noted. 

Y141 is highly conserved both in Keap1 and other KLHLs in various species. Keap1 phosphorylated by Y141 forms Nrf2-associated homodimers and is actively involved in Nrf2 ubiquitination and degradation. Phosphorylation/dephosphorylation of Y141 controls the stability/degradation of Keap1. Residue S104 of Keap1 is important for dimerization, indicating that the disruption of the Keap1 dimer is associated with Nrf2 release. It is almost ubiquitously conserved in KLHL proteins, as well as in the related family of BTB-containing zinc finger transcription factors [38]. Mutation of this serine, which is located in the central region of the BTB domain, is known to disrupt the dimerization potential of BTB [38]. 

Q163 in Keap1 is important for the catalytic activity of the ubiquitin ligase complex [38] and is also found in the vast majority of KLHLs. The motif responsible for the Cullin interaction is called the φ-xE motif and is also shared by the whole KLHL family (and, therefore, could yield another potential concentration-dependent way of their cross-regulation). In human Keap1, the large hydrophobic residue labeled φ is M115, the charged/polar “x” residue is S116, and the conserved glutamate is E117. This last residue is found in 83% of human KLHL proteins and can be replaced by aspartate.

## 4. Discussion

### 4.1. Conserved, Nonconserved, and Partially Conserved Cysteines in KLHLs Contributing to the “Cysteine Code”

Amino acid residues that do not affect the protein function are unlikely to be preserved during evolution, except for very short timescales, due to random mutations, which will be neutral and, hence, would not be eliminated by natural selection. Thus, the conservation of the residue on a large timescale (which, in the case of the KLHL superfamily, at least covers all vertebrate evolution) definitely indicates the important role of this residue in the protein, either structural or functional. However, even more information could be obtained from the analysis of residue conservation both in a single protein (e.g., Keap1) from different species and in a broader context of the superfamily (in our case, KLHLs). This dual approach allows us to distinguish between different reasons for residue conservation. If a residue is conserved both in individual proteins and in the superfamily, it likely influences the protein function in a general way, e.g., it can be part of the catalytic active center (like the Q163 residue in human Keap1, which is important for the catalytic activity of the ubiquitin ligase complex) or participate in an important structural motif (like the double glycine repeats in the DGR domain of Keap1). However, if a certain residue is conserved only in a particular protein family but lacks conservation on a level of a superfamily, it should still be considered functionally important due to its more specific role.

The total number of cysteines in the sequence potentially responsible for redox-dependent inactivation among human proteins is highest in KLHL19 (Keap1) (27) and lowest in KLHL30 and KLHL34 (12 and 13 cysteines, respectively). Two of the three most important regulatory cysteines (C273 and C288) lie outside the major known functional domains (BTB, BACK, and DGR), and the corresponding regions could be aligned only at the level of Clade 2 containing Keap1. Interestingly, in all other members of this clade, these cysteines are absent (Figure 3, Table 1). At position C151, which is located at the end of the conserved domain of BTB, only the KLHL26 protein also has a cysteine, while this position is usually occupied by asparagine. Some cysteine residues involved in human Keap1 regulation are generally located in poorly conserved regions at the N- and C-terminus of Keap1: these include C23, C38, C613, C622, and C624. Our data, however, indicate that these regions are sufficiently conserved only in mammalian Keap1 and are significantly shortened in reptile, fish, and amphibian Keap1 proteins.

The NTR region contains C13, C14, and C38, as well as the important C23 cysteine that is involved in intermolecular dimerization. These cysteines are located in a region that is not aligned with other KLHL proteins and, thus, do not correspond to any residues in them.

The BTB domain contains one of the most reactive of human Keap1 cysteines, C151, which is, however, not conserved in other KLHL proteins (except KLHL26). The basic amino acids that determine its high reactivity (namely, H129, K131, R135, K150, and H154) are localized in spatial proximity to it [21,38,48] and are also missing in other members of the KLHL family. However, several conserved cysteine residues are shared by Keap1 and most other KLHL proteins. The most conserved of these is C171 (it is absent in KLHL25 and KLHL42 only).

The IVR region (positions 185–314, covering the BACK domain and a linker region) is rich in cysteines and contains the most reactive Keap1 cysteines (C257, C273, C288, and C297), which are its major redox sensors [38,47,62,97]. Other KLHL proteins, in general, lack these cysteines. Only KLHL36 has cysteines in proximity to the position corresponding to C288; however, the region is aligned poorly. The same is true for C273 in Keap1 (it may have a corresponding cysteine in KLHL40 only). C257 is conserved in Keap1 only. 

Conversely, although human Keap1 contains the most cysteines of all human KLHLs, a cysteine corresponding to position 184 in Keap1 is present almost everywhere but is absent from Keap1 itself. Interestingly, this cysteine belongs to the 3-box sequence (residues 181–212) responsible for the interaction with Cul3 in the proximal part of the IVR domain [15]. The 3-box sequence motif is common for all BTB adapter proteins [98]. It forms a two-helical hairpin located at the beginning of the BACK domain next to the BTB domain and plays a critical role in the high-affinity interaction with Cul3 [98]. Cysteine C196, belonging to the same sequence, is present in half of the KLHL proteins (all of Clade 2, including Keap1, and half of Clades 3 and 4) but is completely absent in members of Clade 1.

The DGR domain plays a key role in KLHL activity. Nevertheless, in contrast to the strongly conserved BTB and BACK domains, a large variability of amino acids can be observed in sequences of blades that form the β-propeller in different KLHL proteins, especially in the first βA strand. However, both the blades and the loops between them are, to a large extent, conserved inside each separate KLHL protein subfamily (see, e.g., how these regions are conserved in Keap1, Figure S4 in Supplementary File S1). This is inconsistent with the suggestion that these regions are poorly conserved because of the accumulation of random substitutions, including nonconservative ones. Such substitutions in nonfunctional regions with a high probability would not affect the structure (and, consequently, the function) of the protein and, therefore, the overall fitness of the organism. Thus, they would not be subject to the cleansing action of the stabilizing selection. DA and BC loops are well-conserved within each separate KLHL protein, but no similarity is observed between the different proteins, and these loop regions vary in length. As each KLHL protein is likely to have its favorable substrate (such as Nrf2 for Keap1), we suggest that these loops should play an important role in the binding of substrate proteins throughout the entire KLHL protein family. In the case of the DGR region, we assume that these regions, which are not aligned globally but are quite conserved within each KLHL subfamily, should not be considered “variable”. The differences between families come from their functions, as the DA and BC loops bind different substrate proteins. Therefore, they could be quite different between families but still be a subject of purifying selection within each family.

In summary, our data suggest that most of the Keap1 regulatory cysteine residues are conserved only between different (and sometimes not all) Keap1 proteins and are not preserved in other members of the KLHL family. Such distinctive patterns of cysteine residues occurring in different KLHL proteins and even in different Keap1 proteins are referred to as the “cysteine code”. A specific pattern of cysteine residues in Keap1 and their general abundance could occur due to the specific requirement of Keap1 to respond to oxidative stress conditions and deactivate to stop Nrf2 blocking. 

### 4.2. Evolution of “Cysteine Code” in Mammals

It is known that the oxidation or chemical modification of some highly reactive cysteine residues promotes the dissociation of Nrf2 from Keap1 and subsequent nuclear translocation (see Section 1.2.4) [99,100,101]. They are crucial in the regulation of Nrf2, and each of them responds to different oxidants, leading to the formulation of the “cysteine code” hypothesis, according to which different inducers modify certain combinations of cysteines to control the Keap1/Nrf2 stress-sensitive response [49]. The pKa value of a Cys residue (represented by the balance between the thiol and thiolate anion) indicates its reactivity [102]. The lower the pKa value at which the formation of the thiolate anion is favorable, the more reactive Cys becomes. The presence of basic amino acids in close proximity to a Cys residue reduces pKa, thereby increasing its reactivity [103,104]. McMahon et al. suggested that the reactivity of C151 in Keap1 occurs due to the presence of five basic amino acid residues (H129, K131, R135, K150, and H154) located in close spatial proximity to C151 [21] (see Figure 2D). 

These five basic amino acids are able to deprotonate the thiol group in C151, thereby lowering its pKa. This causes the C151 thiol group to exist as an anion under physiological pH conditions. Indeed, the authors showed that Keap1 carrying the K131M, R135M, and K150M triple mutations loses the ability to sense electrophiles that specifically target C151 [21]. The crystal structure of the BTB domain of Keap1 with triterpenoid CDDO was solved in 2014 (PDB (https://www.rcsb.org/, (accessed on 14 April 2023)) ID: 4CXI). The distances between the five mentioned positively charged amino acids that were close to C151 show that R135 is closest to C151 at 3.6 Å between the guanidinium group of Arg and the thiol group of Cys [37], confirming the findings of McMahon et al. [21]. 

The structural features of the Keap1 “cysteine code” in mammals can also be noted. Thus, out of 27 cysteines, only 9 (one-third) have a basic amino acid in their neighbors (Figure 3, Table 1). At the same time, four of them are located in a narrow (~70 amino acids long) band of the linker region between the BACK and the DGR domains. Another one (the most important, C151) is located in the BACK domain. Two more such cysteines (C23 and C38) are located in the N-terminal region (NTR), and one more (C613) is located almost at the very end of the protein in the C-terminal domain. We can assume a late specialization of these residues in the course of protein evolution that required such different changes in reactive amino acids, which, moreover, lie outside the main domains of the protein.

Although the arrangement of cysteines and the surrounding amino acids (the “cysteine code”) is extremely conserved in mammals, the differences that exist nevertheless allow us to attempt to reconstruct the evolution of the “cysteine code” in mammals (see Supplementary File S2 for an analysis of residue conservation and variations for human proteins).

Interestingly, another cysteine with a flanking basic amino acid (C14C15R16) appears in humans as well as in their closest relatives, chimpanzees (*Pan troglodytes*). Moreover, even in their close relative, the orangutan *Pongo abelii* (belonging to the same taxon Hominoidea), after the loss of one of the cysteines, the second one has no basic amino acid (A12C13S14) in its neighbors. In many mammals (for example, other representatives of the clade Euarchontoglires (mice and rats), as well as in some Laurasiatheria (pigs), both cysteines (C13 and C14) are lost. We observe a different picture in close proximity (C23). Indeed, only in mice and rats does this cysteine have the basic amino acid (K22C23P24) in its neighbors, whereas Hominoidea (including humans) have Q22C23P24 at this site, whilst pigs retain neighbors, but the cysteine itself (K22R23P24) is lost.

### 4.3. Modulating Keap1-Nrf2 Interaction Could Have Possible Therapeutic Applications

The Nrf2 protein and its negative regulators Keap1 and Gsk3/β-TrCP act together as a cytoprotective mechanism that allows cells to overcome electrophilic and oxidative stress. Research has shown that manipulating this system by modulating the Keap1-Nrf2 interaction, either through the inhibition of the protein–protein interaction or the covalent modification of Keap1, can provide a powerful therapeutic strategy for a range of diseases [6,105]. 

Gsk3 can be considered a characteristic member of aging programs because, unlike Nrf2, Gsk3 activity increases with age and in pathologies associated with aging, such as type 2 diabetes mellitus, cancer, and neurodegenerative diseases. In addition, Gsk3 is involved in cell death and inflammation (for a review, see [10]). At the same time, the situation of Keap1 (as with KLHLs, in general) is not so unambiguous. The role of KLHLs appears to be in maintaining a balance between cellular proliferation and oncostability. In almost half of the KLHL proteins, the highest level of expression is associated with tissues with a low level of proliferation: the brain and skeletal muscles [69]. Their main function is likely associated with the suppression of proliferation and oncogenesis, including the suppression of protective cell systems (as Keap1 does for Nrf2). Although Keap1 has a wide range of expression, it is also active in the skeletal muscle [69]. The role of Keap1 is attributed mainly to the function of the Nrf2 repressor and redox sensor. However, due to its unusual structure and reactivity, Keap1 undergoes numerous post-translational modifications and has a very rich interactome; thus, it could be active in various cellular processes. It should be noted that active Keap1 is also observed in animal species that do not contain the Keap1-binding domain in the Nrf2, such as *Drosophila melanogaster* or *Caenorhabditis elegans* [13]. This indicates its significance not only as an inhibitor of Nrf2 but as a regulator of proliferation, including by regulating the degradation of proteins other than Nrf2 (p62, Bcl-xL, DPP3, SFRS10, DBP, etc.). 

### 4.4. Nonrandom Positioning of the Cysteines in Keap1?

The position of the relatively numerous cysteines in the DGR domain, which occupies almost half of the protein, and the C-terminus do not coincide in different members of the family. Moreover, the position of cysteines in the Keap1 sequence appears to not be random but at a multiple of approximately 7–7.5 amino acid residues. Of course, this assumption about the location of cysteines requires further research. Indirect evidence for this is the discovery of an additional Keap1 cysteine corresponding to Y208 in human Keap1 in the laboratories of I. Shams and H. Schmidt in long-lived and, at the same time, resistant to hypoxia species: the mole-rat *Spalax ehrenbergi* and the sperm whale, as well as the hibernating hedgehog [106,107]. We refer to this cysteine as CCC (as a designation for a cysteine appearing between two cysteines) and, at the same time, in honor of the representatives of the Department of Evolutionary and Environmental Biology (Mount Carmel, Haifa)—“Shams-Chaban cysteine”. Recently, they drew our attention to the importance of the appearance of this additional cysteine in the already abundant set of cysteines in mammals (28th for humans and 26th for rodents)). Interestingly, this cysteine is a part of the important functional 3-box sequence (residues 181–212) mentioned above, which is present in all KLHL proteins and is responsible for the interaction with Cul3 in the proximal part of the IVR domain [15]. Unexpectedly, this cysteine, absent from human Keap1 (KLHL19), was found in KLHL8, KLHL23, KLHL28, and KLHL40 (Figure 3).

### 4.5. KLHL Family Proteins from Long-Living Species Show No Special Features

Biogerontology studies the interaction of aging and antiaging programs that determine survival curves and their development within the course of evolution. Life span is a stable species characteristic, and its mechanisms must be at least partially programmed in the genome [108,109]. Despite this, longevity is not a primary evolutionary “task” for living organisms. The most important problems of adaptation to the environment are related to survival and reproduction. Longevity could be supported by selection (for example, as “longevity assurance genes” can be associated with some adaptive traits, they are fixed in the population), and because of this, organisms have learned to develop special protective and reparative systems that slow down aging [2,4,5,108,110,111,112]. The efficiency of maintaining homeostasis and repair systems decreases with age [2,4,5,10,108,109,110,113]. Protective systems are under the control of inhibitors that play the role of the representatives of the aging program both directly and through the regulation of biorhythm proteins (circadian and others), i.e., through the master biological clock [10]. The study of proteins located at the intersection of signaling and regulatory pathways and their comparison using biochemical and bioinformatic methods in short- and long-lived species makes it possible to identify the molecular mechanisms underlying the processes and phenomena that regulate ontogenesis timing (including longevity, acute and chronic phenoptosis, the presence of neotenic signs, etc.) [109,113,114,115], as well as, e.g., the potential suppression of the “cytokine storm” in COVID-19 [116]. In this regard, we specifically included in our analysis the KLHL proteins of the blue whale and the naked mole-rat, known for their unusually long lifespans. However, we did not find any specific differences in their KLHL proteins compared to proteins from other species. Perhaps this is due to the rather small number of vertebrate organisms that we considered, and a broader view of the problem is required.

### 4.6. Study Limitations

First, this study suffers from the general limitations of sequence-based analysis, the most important of which is the hypothetical nature of the conclusions made. In our case, we could only assume that a certain residue is important in some sense, but experimental verification of its predicted function is still required. Second, we restricted ourselves to the analysis of vertebrate KLHL proteins, thus leaving aside the entire evolutionary history of these proteins prior to the appearance of vertebrates. Third, even inside the vertebrates we covered, only a small sample of complete genomes was analyzed; thus, further research is required to identify positions where additional cysteine residues emerged.

## 5. Conclusions

The identification of the specificity of related proteins and the search for new binding sites is an urgent problem for modern pharmacological and biogerontological research [117]. In order to summarize and generalize the data accumulated for Keap1, we compared sequences of Keap1 and its homologs from several major groups of vertebrates. This approach allows us to determine whether the previously established functional properties of a residue or a whole region of the protein are specific to that particular protein or if they have general importance. For example, it is known that, despite the fact that the Keap1 protein already exists in the most primitive Metazoa, it is not involved in the inhibition of the activity of the Nrf2 transcription factor, as the Nrf2 analog (for example, in *D. melanogaster* and *C. elegans*) does not contain a domain for Keap1 interaction.

### 5.1. Development of the “Cysteine Code” Is an Evolutionary Strategy of Keap1

An important characteristic of Kelch-like proteins is the “cysteine code” (a location of cysteine residues in various members of the KLHL family). It is unique for each member of the family. Amino acids adjacent to a cysteine residue also play important roles and can be considered part of the “cysteine code”. Summing up the consideration of the “cysteine code”, we can assume an adaptation of the Keap1 protein to the role of a redox sensor. 

In Clade 2, the number of differences from a consensus sequence among 275 amino acids which we have considered conserved on the multiple alignments is the smallest in KLHL20 (56 substitutions) and much larger in Keap1 (about 100, which is quite a lot for Clade 2).

Thus, being the most important representative of Clade 2, Keap1 is, nevertheless, located on its periphery. It is interesting that not only does the amount of its cysteines exceed that of any other member of the family (in humans), but that, at the same time, their location suggests their relatively late appearance in the course of evolution. They are predominantly not allocated in the conserved regions (for example, BTB and BACK) of domains but rather in the IVR region outside the BACK domain, as well as in the N- and C-terminal regions, thus, generally outside the functional domains.

### 5.2. Generally, Nonconserved Cysteine Residues May Be Pivotal for Keap1 Regulation

Two of the most active cysteines (C273 and C288) of Keap1 proved not to be essential for Keap1′s ability to suppress Nrf2 accumulation (mutants with C273M or C273W and either C288E, C288N, or C288R retained this ability) [48]. These residues are prone to the formation of crosslinks or the influence of any of the chemicals; thus, their role in Keap1 is solely regulatory. It is important that, in the IVR region, out of the three most important (the most functionally active) cysteines, only C297 is conserved in other KLHL proteins, while C273 and C288 are found in Keap1 only. 

We identified several cysteine residues that are at least partially conserved in Keap1 but are generally missing in other members of the KLHL family (they are marked with the green color in Table 1). These residues include the aforementioned C273 and C288, as well as C13, C14, C23, C38, C151, C226, C241, C249, C257, C368, C395, C406, C434, C518, C613, C622, and C624. We suggest that these residues could play an important regulatory role in Keap1-Nrf2 interaction, and they are worth further experimental study. To our knowledge, at least some of them have not been the subject of such studies before (C368, C395, C406, and C518).

### 5.3. Predicting Malevolent Mutations in Keap1 That Could Increase or Decrease Its Sensitivity towards Oxidative Stress Conditions

It is known that cysteine residues in the Keap1 molecule interact with alkylating agents and pro-oxidants at different rates, and this occurs because the pKa value is dependent on their amino acid environment. It is hypothesized that for a cysteine-dependent biosensor, such as Keap1, mutations that add cysteines (S243C, G333C, and R470C) or those that lower the relative pKa of existing cysteines (e.g., by adding basic residues into their immediate vicinity) make them more susceptible to electrophilic attacks [21] and facilitate Keap1 inactivation and, consequently, Nrf2 activation.

Interestingly, in KLHLs, overconservative positions often emerge in the close proximity of cysteines (in the BTB domain, these are C77/D78, C95/H96, A101/C102 (S102 in Keap1), C103 (S103 in Keap1)/S104, and C151/V152; in the IVR domain, these are C241/E242, A248/C249, D256/C257, and R272/C273; and in the DGR domain, these are C406/A407and G433/C434; see Table S2 in Supplementary File S2 for further details).

Based on our findings, we can suggest that mutations that remove cysteine residues from Keap1, especially the most reactive ones and, therefore, those involved in the recognition of oxidative stressors (see Section 1.2.3), would cause the constant inhibition of Nrf2. A similar but less pronounced effect could be caused by the replacement of the basic residues flanking them, and, *vice versa*, mutations that would add basic residues to the close proximity to these cysteines would facilitate Keap1 inactivation and, consequently, Nrf2 activation. The corresponding cysteines can differ in reactivity even in relatively closely related species (for example, in humans and mice). Thus, a cysteine in the same position of Keap1 could be “strong” in one species and “regular” in another, and, accordingly, proteins from different species would have different abilities to regulate the activity of the Nrf2 transcription factor. Analysis of the conservation of corresponding amino acids in Keap1 and among other KLHL proteins allows us to predict which properties of cysteine residues could be transferred from, for instance, murine Keap1 to the human protein. This could be important for the development of specific Keap1 inhibitors.

### 5.4. Applications of Identifying New Members of the KLHL Protein Family

The human genome encodes 42 members of the KLHL family, which contains conserved BTB, BACK, and DGR domains. Our analysis revealed that some proteins assigned to another family, KBTBD (namely the KBTBD2, KBTBD3, KBTBD4, KBTBD6, KBTBD7, KBTBD8, KBTBD12, and calicin proteins), contain all the same essential domains and can be considered on par with the already recognized members of the KLHL family. KLHL proteins are known to be involved in the ubiquitination process, but the specific roles of each member of the KLHL family vary, as do their substrates. The cross-dimerization of BTB between different KLHL proteins could theoretically allow for different substrate binding depending on the spatial and temporal expression of the KLHL proteins [19]. SCF-FBXL17 is an E3 dimerization quality control ligase, which ubiquitylates and helps cleave inactive BTB protein heterodimers while maintaining functional homodimers. SCF-FBXL17 was found to degrade aberrant BTB dimers that fail to stabilize the intermolecular β-sheet around the highly divergent β-strand of the BTB domain [118].

Therefore, the recognition of substrates and corresponding biological functions for each KLHL member will not only provide clues to the mechanism of pathogenesis but also facilitate targeted therapies for related diseases. For example, if interacting functional KLHL proteins are found to contribute to the course of a disease, a small molecule can be designed to indirectly enhance the ubiquitin-dependent degradation of substrates by inhibiting the appropriate deubiquitinating enzymes. In contrast, when substrates are involved in the maintenance of normal physiological functions and disease resistance, a small molecule can be engineered to slow down the process of proteasome degradation by targeting the E3 Cul3-KLHL ligases. Moreover, as the network of relationships between KLHL protein functions and associated diseases is built, KLHL is likely to act as a marker of prognostic or diagnostic differences in cancer and hereditary diseases. A similar phenomenon (the appearance of the alternative splicing of Nrf2 without a region corresponding to exon 2 and, therefore, incapable of interacting with Keap1) occurs in tumor cells [119,120]. Mutations in some KLHL genes lead to the development of pathologies, including inherited ones. Hopefully, in the future, the use of drugs that regulate their activity can not only be used to treat neurodegenerative pathologies but also to increase healthy life expectancy and longevity. The ability to suppress the activation of the expression of protective cell enzymes is controlled by Nrf2. Additionally, as the three-dimensional structure of more KLHLs is analyzed, an increasing number of potential inhibitors can be engineered for clinical treatment. A deeper understanding may be important for the development of new inhibitors and activators, as well as for determining optimal use for targeted protein degradation.

## Figures and Tables

**Table 1:** Conservation of cysteine residues in human Keap1. Residues whose modifications were experimentally studied before (see Section 1.2.4) are marked in the last column with the + sign. Most strong effectors are shown in bold. The Cys residues mostly conserved in Keap1 (at least on the clade level) are shown in blue. The mainly conserved Cys residues (at least at the clade level) that are absent in Keap1 are shown in red. The green color shows nonconserved Cys residues that exist in Keap1 only. The asterisk sign * marks cysteine residues that have neighboring basic amino acid residues.

Domain	Residue, Number	Conserved in Keap1	Conserved in Clade 2	Conserved in KLHLs	Comment	Studied
NTR	C13	No	-	-		+
NTR	C13	No	-	-		+
NTR	C14 *	No	-	-		+
NTR	C23	No	-	-		+
NTR	C38 *	Yes	-	-	Except for Keap1a	+
BTB	C77	Yes	Yes	Partially (1,3)		+
BTB	C95	-	No	Partially (1,4)		
BTB	101–104	In this region, many KLHL proteins have cysteines but not Keap1				
**BTB**	C151 *	**Yes**	**No**	**No**		+
BTB	C160	-	Partially	No		
BTB	C171	Yes	Yes	Yes		
BTB	C172	-	Yes	Partially		
BACK	C184	-	Yes	Yes		
BACK	C188	-	No	Partially	Rarely occurs in different clades	
BACK	C196	Yes	Yes	Partially (1,2,4)		
BACK	206–208	In this region, many KLHL proteins have cysteines but not Keap1				
BACK	C226 *	Yes	No	No		+
BACK	C241 *	Yes	No	No		+
BACK	C249	Yes	No	No		+
BACK	C257	Partially	No	No	Except for Keap1a and Keap1 in fish	+
**IVR**	C273 *	**Yes**	**No**	**-**	**Except for Keap1a**	+
**IVR**	C288 *	**Yes**	**No**	**-**	**Except for Keap1 in fish**	+
IVR	C297 *	Yes	Yes	-		+
IVR	C319 *	Partially	-	-	In mammals only	
Kelch1	C344	-	Partially	-		
Kelch2	C366	-	No	Partially (3)		
Kelch2	C368	Yes	No	No		
Kelch2	C395	Yes	Partially	No		
Kelch2	C406	Yes	No	No		
Kelch3	C434	Yes	No	-		+
Kelch3	C442	-	No	Partially (1,3)		
Kelch4	C489	Yes	No	Partially		
Kelch5	C513	Yes	No	Partially	Except for Keap1a	
Kelch5	C518	Partially	No	No		
Kelch5	C536	-	No	Partially		
Kelch6	C583	Yes	No	Partially		
CTR	C613*	Yes	-	-		+
CTR	C622	Partially			Missing in *Xenopus*	+
CTR	C624	Partially			Missing in *Xenopus*	+

**Table 2:** Conservation of the Nrf2-binding residues in the KLHL family.

Loop Location	Residue, Number	Binds to	Conserved in Keap1	Conserved in Clade 2	Conserved in KLHLs
DA (1→2)	S363	ETG**E**	Yes	No	-
BC (2)	R380	ETG**E**	Yes	-	-
BC (2)	N382	ETG**E**	Yes	-	-
DA (2→3)	N414	**D**IDLG	Yes	No	No
DA (2→3)	R415	DI**D**LG **E**TGE	Yes	No	No
BC (4)	R483	**E**TGE	Yes	-	-
DA (4→5)	S508	**E**TGE	Yes	Partially	-

## Data Availability

The data presented in this study (multiple sequence alignment of KLHL proteins including Keap1) are openly available in ResearchGate at [https://doi.org/10.13140/RG.2.2.10252.18566].

## Supplementary Materials

The following supporting information can be downloaded at: https://www.mdpi.com/article/10.3390/life13041045/s1. The following files are enclosed in the paper: (1) Supplementary_File S1 Contains: Table S1. The 50 human proteins studied in this paper. Figure S1. Phylogenetic tree of closest relatives of leucine-zipper-like transcription transcription regulator 1 (LZTR1, NP_006758.2, or LZTR1_HUMAN). Figure S2. Phylogenetic tree of the KLHL proteins with the domain structure mapped onto it. Figure S3. Short version of the phylogenetic tree of the KLHL proteins with several important residues. Figure S4. Multiple alignments of Keap1 proteins from genomes of nine selected *Vertebrata* species. Figure S5. Short version of the phylogenetic tree of the KLHL proteins with Nrf2 binding site. Figure S6. Comparison between domain borders produced by the Pfam profile HMM search (top) and those annotated in the UniProt/SwissProt database (bottom) for human Keap1 protein. (2) Supplementary_File S2 Contains: Table S2. Detailed description of conservation of cysteines and other functionally and structurally important residues in Keap1 (KLHL19) and other members of the KLHL family.

## Abbreviations

DGR—double glycine repeat; Gsk3—glycogen synthase kinase 3; Nrf2—nuclear factor erythroid 2-related factor 2; Keap1—Kelch-like ECH-associated protein 1; KLHL–Kelch-like; ROS—reactive oxygen species; β-TrCP—β-transducin repeat-containing protein.
