# Supplementary material for: Regulation of Cell Proliferation and Nrf2-Mediated Antioxidant Defense: Conservation of Keap1 Cysteines and Nrf2 Binding Site in the Context of the Evolution of KLHL Family
Source: Life (Basel). 2023 Apr 19;13(4):1045. doi: 10.3390/life13041045 (PMC10146909; doi:10.3390/life13041045)

Supplementary file 1

to the article: "Regulation of Cell Proliferation and Nrf2-Mediated Antioxidant Defense: conservation of Keap1 cysteines and Nrf2 binding site in the context of the evolution of KLHL Family".

Table S1*. 50 human proteins which were studied in this paper**. Sorting of proteins is done according to the phylogenetic tree in ***Figure 3 of the main text.

Protein ID	Clade	Name	UniProt ID	Other names
NP_001243503.1	3	KLHL37	ENC1_HUMAN	Ectoderm-neural cortex protein 1 (ENC1)
NP_071925.2	3	KLHL25	KLH25_HUMAN	Ectoderm-neural cortex protein 2 (ENC2)
NP_001186219.1	3	KLHL23	KLH23_HUMAN	
NP_055666.2	3	KLHL21	KLH21_HUMAN	
NP_940984.3	3	KLHL30	KLH30_HUMAN	
NP_443152.1	3	KLHL29	KLH29_HUMAN	
NP_001075144.2	3	KLHL38	KLH38_HUMAN	
NP_569713.2	3	KLHL6	KLHL6_HUMAN	
NP_001034637.2	3	KLHL35	KLH35_HUMAN	
NP_060114.2	3	KLHL24	KLH24_HUMAN	Kainate receptor-interacting protein for GluR6 (KRIP6)
NP_940841.1	3	KBTBD3	KBTB3_HUMAN	
XP_006718324.1	3	KBTBD4	KBTB4_HUMAN	
NP_689606.2	4	KLHL40	KLH40_HUMAN	Sarcosynapsin
NP_006054.2	4	KLHL41	KLH41_HUMAN	Sarcosin
NP_997218.2	4	KBTBD12	KBTBC_HUMAN	
NP_056298.2	4	KBTBD2	KBTB2_HUMAN	
NP_115894.2	4	KBTBD8	KBTB8_HUMAN	T-cell activation kelch repeat protein (TA-KRP)
NP_690867.3	4	KBTBD6	KBTB6_HUMAN	
NP_115514.2	4	KBTBD7	KBTB7_HUMAN	
XP_005257330.1	2	KLHL10	KLH10_HUMAN	
NP_036421.2	2	KLHL19	KEAP1_HUMAN	Kelch-like ECH-associated protein 1 (Keap1)
NP_005888.1	2	KLHL27	IPP_HUMAN	Actin-binding protein IPP
XP_006715816.1	2	KLHL7	KLHL7_HUMAN	
XP_005267826.1	2	KLHL28	KLH28_HUMAN	
XP_005262711.1	2	KLHL5	KLHL5_HUMAN	
NP_065917.1	2	KLHL1	KLHL1_HUMAN	Mayven-related protein 2 (MRP2)
NP_476503.1	2	KLHL4	KLHL4_HUMAN	
NP_065854.3	2	KLHL8	KLHL8_HUMAN	
XP_005265058.1	2	KLHL18	KLH18_HUMAN	
XP_005245460.1	2	KLHL12	KLH12_HUMAN	
XP_006711340.1	2	KLHL20	KLH20_HUMAN	Kelch-Like ECT2 Interacting Protein (KLEIP)
XP_006710663.1	2	KLHL17	KLH17_HUMAN	Actinfilin
NP_059111.2	2	KLHL3	KLHL3_HUMAN	
NP_001154993.1	2	KLHL2	KLHL2_HUMAN	Actin-binding protein Mayven
NP_071324.1	5	KLHL16	GAN_HUMAN	Gigaxonin
NP_006460.2	5	KLHL39	NS1BP_HUMAN	Influenza virus NS1A-binding protein (IVNS1ABP)

NP_005884.2	5	KBTBD14	CALI_HUMAN	Calicin
NP_060613.1	5	KLHL11	KLH11_HUMAN	
XP_005267404.1		KLHL33	KLH33_HUMAN	
NP_443136.2	1	KLHL32	KLH32_HUMAN	
NP_060786.1	1	KLHL26	KLH26_HUMAN	
XP_006724405.1	1	KLHL22	KLH22_HUMAN	
NP_079007.2	1	KLHL36	KLH36_HUMAN	
NP_001161771.1	1	KLHL13	KLH13_HUMAN	
NP_061335.1	1	KLHL9	KLHL9_HUMAN	
NP_001003760.2	1	KLHL31	KLH31_HUMAN	
NP_085127.2	1	KLHL15	KLH15_HUMAN	
XP_006722575.1	1	KLHL14	KLH14_HUMAN	Protein interactor of Torsin-1A (Printor)
NP_695002.1	1	KLHL34	KLH34_HUMAN	
NP_065833.1	1	KLHL42	KLH42_HUMAN	Cullin-3-binding protein 9 (Ctb9)

Figure captions:

***Figure S1*. Phylogenetic tree of closest relatives of leucine-zipper-like transcription regulator 1 (LZTR1, NP_006758.2 or LZTR1_HUMAN)**. According to our domain analysis, it can be considered as a member of the KLHL family. All proteins have the kelch-repeat domain on the N-terminus. 34 manually inspected proteins were aligned with Muscle (Edgar, 2004) and the tree for them was constructed in MEGA7 with the neighbor-joining algorithm (Kumar, et al., 2016). Numbers on the branches are the bootstrap test support calculated for the 100 replicates. All positions with less than 90% site coverage were eliminated, leaving 455 positions for the tree construction. Tree leaves are colored according to the taxonomy of the respective organisms. Domain structure was obtained with Domain Analyzer software (https://depo.msu.ru/module/domainanalyser) and Pfam database (Finn, et al., 2016).

***Figure S2*. Phylogenetic tree of the KLHL proteins with their domain structure mapped onto it.** 449 proteins sampled as described in the Materials and Methods section were aligned with Muscle (Edgar, 2004) and the tree for them was constructed in MEGA7 with the neighbor-joining algorithm (Kumar, et al., 2016). Numbers on the branches are the bootstrap test support calculated for the 100 replicates (values lower than 10 are not shown). We used manually selected regions (conserved blocks) consisting of 202 positions for the tree construction. Domain structure was obtained with Domain Analyzer software (https://depo.msu.ru/module/domainanalyser) and Pfam database (Finn, et al., 2011; Finn, et al., 2016). Human proteins are shown in blue color for known members of the KLHL family and in red color for proteins previously considered to belong to the KBTBD family.

Figure S3*. Short version of the phylogenetic tree of the KLHL proteins with several important residues** (see ***Figure S2 for the full version). Clades containing human proteins are collapsed to a single sequence. Positions which are known to be important for the structural integrity and function are mapped on the tree.

Figure S4*. Multiple alignment of Keap1 proteins from genomes of 9 selected *Vertebrata* species**. Residues which are marked in the ***Figure 3 on the main text are marked in the `CYS_IMPORTANT_RESIDUES` row (X marks positions poorly aligned between different KLHL proteins, C marks aligned positions). Structurally important residues which are depicted in ***Figure S3*** are marked in the `STRUCTURE_AND_FUNCTION` row. Regions where good multiple alignment exists between different KLHL proteins and which were chosen for the phylogenetic tree construction are marked in the `BLOCKS` row. Domain boundaries as predicted by Pfam domain models for human sequence are given in the `FORMULA` row. Alignment was visualized in GeneDoc software (http://nrbsc.org/gfx/genedoc).

Figure S5*. Short version of the phylogenetic tree of the KLHL proteins with Nrf2 binding site** (see ***Figure S2 for the full version). Clades containing human proteins are collapsed to a single sequence. Nrf2 binding site positions are mapped on the tree.

Figure S6. **Comparison between domain borders produced by the Pfam profile HMM search (top) and those annotated in the Uniprot/SwissProt database (bottom) for human Keap1 protein**. Search for the corresponding Pfam domains yields the following domain coordinates: BTB [67–178], BACK [185–285], Kelch1 [324–358], Kelch2 [361–410], Kelch3 [412–456], Kelch4 [459–504], Kelch5 [507–550], Kelch6 [553–597]. Uniprot annotation for the same protein (KEAP1_HUMAN) provides the following coordinates: BTB [77–149], BACK [184–286], Kelch1 [327–372], Kelch2 [373–423], Kelch3 [424–470], Kelch4 [471–517], Kelch5 [518–564], Kelch6 [565–611]. The corresponding regions were mapped onto the AlphaFold model of Keap1 protein (provided in the Uniprot database): **(A)** BTB domain is shown in orange, BACK domain is shown in magenta. **(B)** Two neighboring blades of β-propeller are shown. The first Kelch domain is colored lime, the last Kelch domain is colored purple.

References:

Edgar, R.C. MUSCLE: a multiple sequence alignment method with reduced time and space complexity. *BMC Bioinformatics* 2004;5:113.

Finn, R.D., Clements, J. and Eddy, S.R. HMMER web server: interactive sequence similarity searching. *Nucleic Acids Res* 2011;39(Web Server issue):W29-37.

Finn, R.D., *et al.* The Pfam protein families database: towards a more sustainable future. *Nucleic Acids Res* 2016;44(D1):D279-285.

Kumar, S., Stecher, G. and Tamura, K. MEGA7: Molecular Evolutionary Genetics Analysis Version 7.0 for Bigger Datasets. *Mol Biol Evol* 2016;33(7):1870-1874.

Figure S1. Phylogenetic tree of closest relatives of leucine-zipper-like transcription regulator 1 (LZTR1, NP_006758.2 or LZTR1_HUMAN)

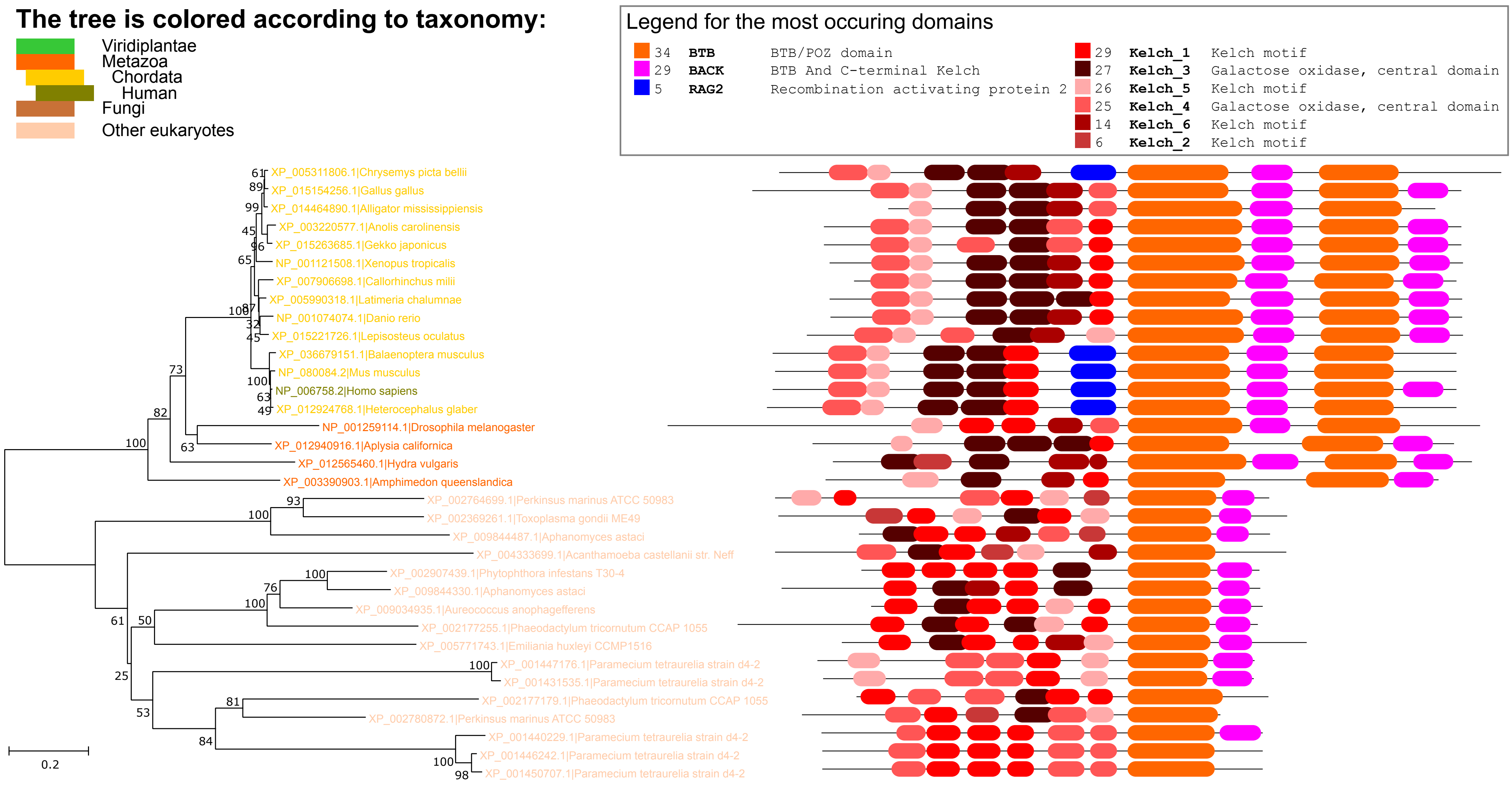

***Figure S2.* Phylogenetic tree of the KLHL proteins with their domain structure mapped onto it**

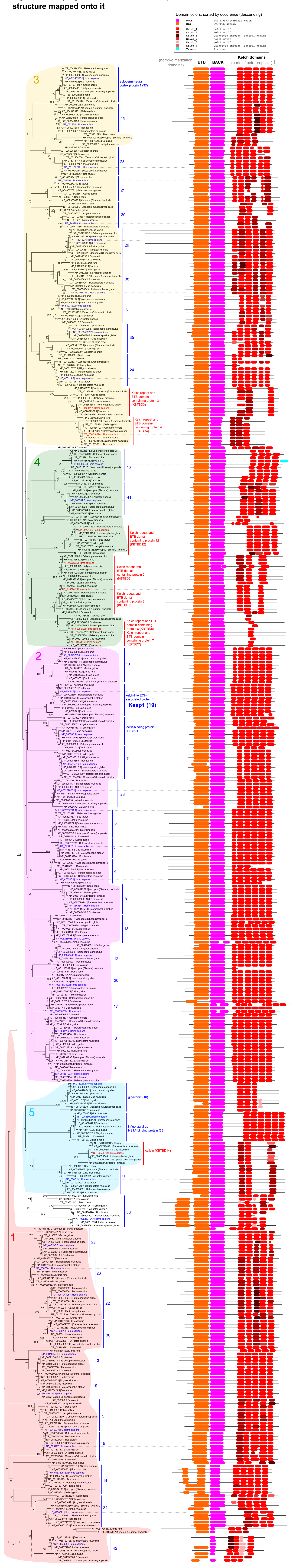

Figure S3. Short version of the phylogenetic tree of the KLHL proteins with functionally important positions

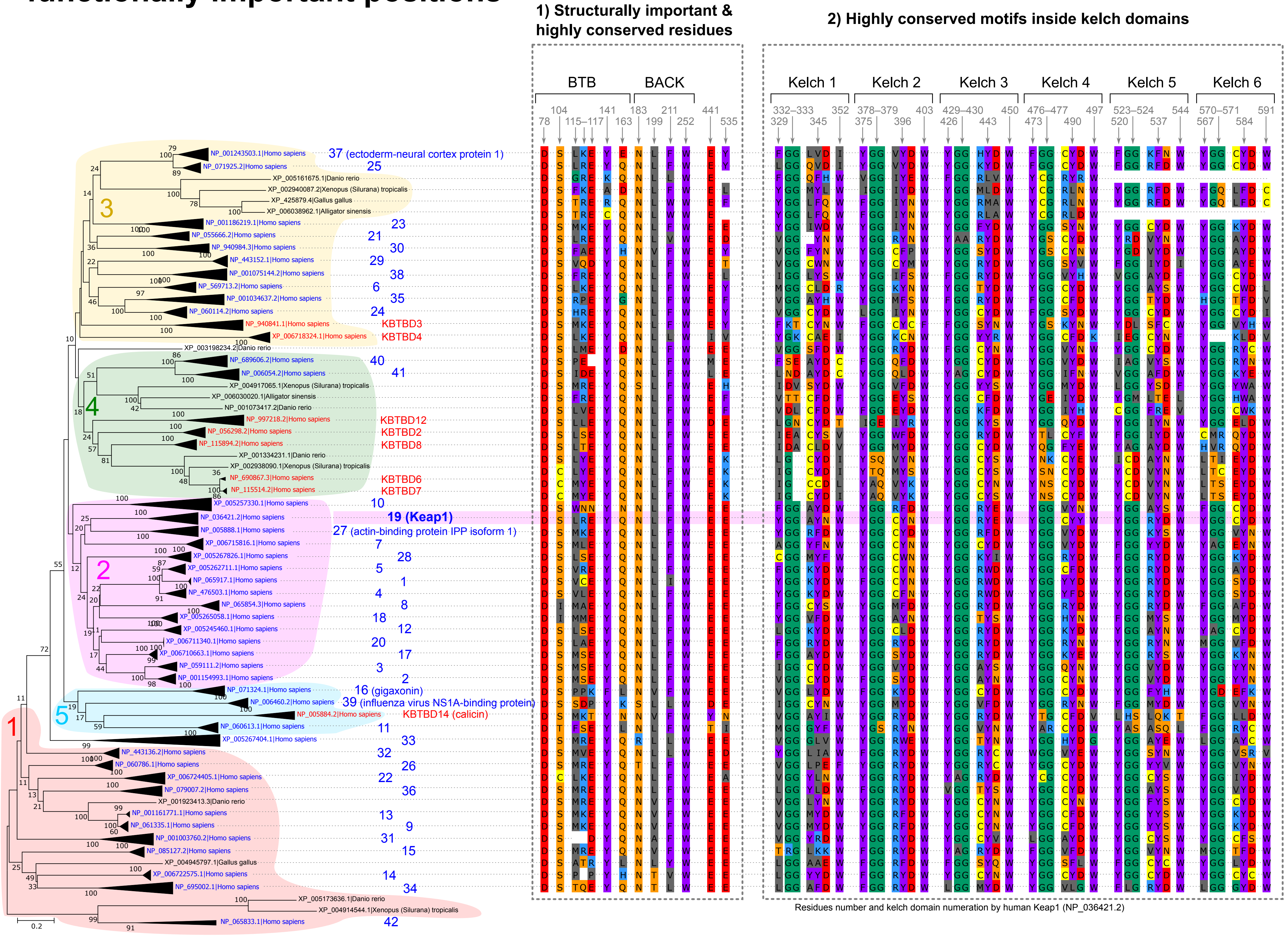

Figure S4*. Multiple alignment of Keap1 proteins from genomes of 9 selected *Vertebrata* species**. Residues which are marked in the ***Figure 3 on the main text are marked in the `CYS_IMPORTANT_RESIDUES` row (X marks positions poorly aligned between different KLHL proteins, C marks aligned positions). Structurally important residues which are depicted in ***Figure S3*** are marked in the `STRUCTURE_AND_FUNCTION` row. Regions where good multiple alignment exists between different KLHL proteins and which were chosen for the phylogenetic tree construction are marked in the `BLOCKS` row. Domain boundaries as predicted by Pfam domain models for human sequence are given in the `FORMULA` row. Alignment was visualized in GeneDoc software (http://nrbsc.org/gfx/genedoc).

```
CYS_IMPORTANT_RESIDUES             : ------------XX--------X--------------X---------------------------------------C-------
STRUCTURE_AND_FUNCTION             : ------------------------------------------------------------------------------Z------
NP_036421.2|Human                  : MQPDPRPSGAGACCRFLPLQSQCPEGAGDAVMYASTECKAEVTPSQ-HGNRTFSYTLEDHTKQAFGIMNELRLSQQLCDVTLQVK
NP_001103775.1|Mouse               : MQPEPKLSGAPRSSQFLPLWSKCPEGAGDAVMYASTECKAEVTPSQ-DGNRTFSYTLEDHTKQAFGVMNELRLSQQLCDVTLQVK
NP_001094612.1|Cattle              : MQPEPRPSGAGAHTQFLPLRSQRPEGAGDTAMYASTECKAEVTPSQ-HGNRTFSYTLEDHTKQAFGIMNELRLSQQLCDVTLQVK
XP_036702860.1|Blue_whale          : MQLEPGPSGAGAHTQFLPLRSQRPEGAGDTVMYASTECKAEVTPSQ-HGNRTFSYTLEDHTKQAFGIMNELRLSQQLCDVTLQVK
XP_004865558.1|Naked_mole_rat      : MQPEPRPSRARARSRFLPLHSECPEGAGDTVMYASTECKAEVTPSQ-DGNRTFSYTLEDHTKQAFGIMNELRLSQQLCDVTLQVK
XP_006023504.1|Chinese_alligator   : -------------------------------MYAP-ECQAEVTPSP-AGPRSFSYTLDEHPCQALAIMNELRLSQQLCDVTLRVR
NP_001008024.1|Western_clawed_frog : -------------------------------MYAT-ECQAEVTPSHSNGNRQFQYSLADHTNQAFQIMNELRLGQQLCDVKLKVK
NP_001106948.1|Danio_rerio         : ----------------------------MLAAAGMTECKAEVTPSASNGHRVFSYTLESHTAAAFAIMNELRRERQLCDVTLRVR
NP_878284.2|Danio_rerio            : -----------------------MICPRKKRPIKDEDFSAIVVPSM-RGHGYLDYTVESHPSKALQNMDELRHHEMLCDLVLHVT
XP_002940707.2|Western_clawed_frog : ----------MALSHAFFNQNSRMLCPRKKKQPCLKNCTAIAVPSM-KGHGYLDYTIENHTSKAFEKMDEMRRNNLLCDVLLRVS
BLOCKS                             : -------------------------------------------------------------------BBBBBBBBBBBBBBB---
FORMULA                            :

CYS_IMPORTANT_RESIDUES             : -----------C-----CCCC-----------------------------------------------C--------C-------
STRUCTURE_AND_FUNCTION             : --------------------Z-----------ZZZ-----------------------Z---------------------Z----
NP_036421.2|Human                  : YQDA-PAAQFMAHKVVLASSSPVFKAMFTN-GLREQGMEVVSIEGIHPKVMERLIEFAYTASISMGEKCVLHVMNGAVMYQIDSV
NP_001103775.1|Mouse               : YEDI-PAAQFMAHKVVLASSSPVFKAMFTN-GLREQGMEVVSIEGIHPKVMERLIEFAYTASISVGEKCVLHVMNGAVMYQIDSV
NP_001094612.1|Cattle              : YQDA-PAAQFMAHKVVLASSSPVFKAMFTN-GLREQGMEVVSIEGIHPKVMERLIEFAYTASISMGEKCVLHVMNGAVMYQIDSV
XP_036702860.1|Blue_whale          : YQDA-PAAQFMAHKVVLASSSPVFKAMFTN-GLREQGMEVVSIEGIHPKVMERLIEFAYTASISMGEKCVLHVMNGAVMYQIDSV
XP_004865558.1|Naked_mole_rat      : YEDI-PTAQFMAHKVVLASSSPVFKAMFTT-GLREQGMEVVSIEGIHPRVMERLIEFAYTASISMGEKCVLHVMNGAVMYQIDSV
XP_006023504.1|Chinese_alligator   : YRDA-PPADFQAHKVVLASSSPVFRAMFTA-GLREQGMEVVPIEGIHPRVMERLIEFAYTASIAVGERCVLHVMNGAVMYQIDSV
NP_001008024.1|Western_clawed_frog : YND--VQDEFVAHKIVLASSSPVFRAMFTN-GLRECGMETVTIEGVHPKVMQRLLEFAYTASISVGEKCVIHVMNGAVMYQMDSV
NP_001106948.1|Danio_rerio         : YCPLDTHVDFVAHKVVLASSSPVFRAMFTN-GLKECGMEVVPIEGIHPKVMGRLIEFAYTASISVGEKCVIHVMNGAVMYQIDSV
NP_878284.2|Danio_rerio            : YKD--KIVDFKVHKLVLAASSPYFKAMFTS-NFKECHASEVTLRDVCPQVISRLIDFAYTSRITVGETCVLHVLLTAMRYQMEEV
XP_002940707.2|Western_clawed_frog : YNG--KEEDFHVHKIVLASCSPFFRAMFTN-NFRECHAREVTIKDICPKVMQRLIEFAYTSRITVGEKCVLHVLLAAMRYQMEDV
BLOCKS                             : ------BBBBBBBBBBBBBBBBBBBBBBBBBBBBBB----------BBBBBBBBBBBBBBBBBB------BBBBBBBBBBBBBBB
FORMULA                            :

CYS_IMPORTANT_RESIDUES             : ---CC-----------C---C-------C---------CCC---------------------C--------------C-------
STRUCTURE_AND_FUNCTION             : ---------------Z---------------Z-----------Z-----------------------------------------
NP_036421.2|Human                  : VRACSDFLVQQLDPSNAIGIANFAEQIGCVELHQRAREYIYMHFGE----VAKQEEFFNLSHCQLVTLISRDDLNVRCESEVFHA
NP_001103775.1|Mouse               : VRACSDFLVQQLDPSNAIGIANFAEQIGCTELHQRAREYIYMHFGE----VAKQEEFFNLSHCQLATLISRDDLNVRCESEVFHA
NP_001094612.1|Cattle              : VRACSDFLVQQLDPSNAIGIANFAEQIGCTELHQRAREYIYMHFGE----VAKQEEFFNLSHCQLATLISRDDLNVRCESEVFHA
XP_036702860.1|Blue_whale          : VRACSDFLVQQLDPSNAIGIANFAEQIGCTELHQRAREYIYMHFGE----VAKQEEFFNLSHCQLVTLISRDDLNVRCESEVFHA
XP_004865558.1|Naked_mole_rat      : VRACSDFLVQQLDPSNAIGIANFAEQIGCTELHQRAREYIYMHFGERPLQVAKQEEFFNLSHCQLATLISRDDLNVRCESEVFHA
XP_006023504.1|Chinese_alligator   : VRACADFLAQQLDPSNAIGIANFAEQIDCSELHQRAREYIYRHFAE----VAKQEEFFNLSHCQLVTLISRDELNVRCESEVFHA
NP_001008024.1|Western_clawed_frog : VKACCDFLIQQLDPSNAIGIATFAEQIGCHELHQKAREYIYMQFGE----VAKQEEFFNLSSCQLVNLVSRDELNVRCESEVFHA
NP_001106948.1|Danio_rerio         : VQACCDFLVEQLDPSNAIGIASFAEQIGCTELHQKAREYIYMNFSQ----VATQEEFFTLSHCQLVTLISRDELNVRCESEVFHA
NP_878284.2|Danio_rerio            : AKACCDFLMKNLEPSNVIGISRFAEEIGCTDLHLRTREYINTHFNE----VTKEEEFFSLSHCQLLELISQDSLKVLCESEVYKA
XP_002940707.2|Western_clawed_frog : AKACSDFLVKHLDASNVIGISNFAEQIGCHELYRTGREYINTHFSE----VTNEEEFLSLSHCELLDLVSQDSLNVLCESEVYNA
BLOCKS                             : BBBBBBBBBBBBBBBBBBBBBBBBBBBBBBBBBBBBBBBBBBBBBBBBBBBBBBBBBBBBBBBBBBBBBBBBBBBBBBBBB---B
FORMULA                            :
```

```
CYS_IMPORTANT_RESIDUES             : C-------C---------------X--------------X--------X---------------------X--------------
STRUCTURE_AND_FUNCTION             : ---Z----------------------------------------------------------------------------Y--GG
NP_036421.2|Human                  : CINWVKYDCEQRRFYVQALLRAVRCHSLTPNFLQMQLQKCEILQSDSRCKDYLVKIFEELTLHKPTQVMPCRAPKVGRLIYTAGG
NP_001103775.1|Mouse               : CIDWVKYDCPQRRFYVQALLRAVRCHALTPRFLQTQLQKCEILQADARCKDYLVQIFQELTLHKPTQAVPCRAPKVGRLIYTAGG
NP_001094612.1|Cattle              : CINWVKYDCEQRRFYVQALLRAVRCHSLTPHFLQMQLQKCEILQSDSRCKDYLVKIFQELTLHKPTQVMPCRAPKVGRLIYTAGG
XP_036702860.1|Blue_whale          : CINWVKYDCEQRRFYVQALLRAVRCHSLTPHFLQMQLQKCEILQSDSRCKDYLVKIFQELTLHKPTQVMPCRAPKVGRLIYTAGG
XP_004865558.1|Naked_mole_rat      : CINWVKYDCEQRRFYVQALLRAVRCHALTPHFLQMQLQKCEILQSDSRCKDYLVQIFQELTLHKPTPAMPCRAPKVGRLIYTAGG
XP_006023504.1|Chinese_alligator   : CIDWVRYDCASRRPYVQALLRAVRCHALSPGFLQLQLRECEVLQGDTRCQDYLAQIFQDLTLHKPTQRLPSRTPKVGQLIYAVGG
NP_001008024.1|Western_clawed_frog : CINWVKYDCENRRPYIQALLRAVRCHSLTPNFLQLQLQRCEILKGDSRCQDYLSQIFQDLTLHKPTLPLQGRIPNVPQFIYVAGG
NP_001106948.1|Danio_rerio         : CVAWVQYDREERRPYVQALLQAVRCHSLTPHFLQRQLEHFEW---DAQSKDYLSQIFRDLTLHKPTKVIPLRTPKVPQLIYTVGG
NP_878284.2|Danio_rerio            : CIDWVRWDAESRAQYFHALLNAVHIYALPPTFLKRQLQSCPILSKANSCKDFLSKIFHEMALRKPLPPTPHRG---TQLIYIAGG
XP_002940707.2|Western_clawed_frog : CLKWMQWDLNNRAQYFHALLNAVHLYSLPAKFLEIQLKKCPILSKENRCRVYLSKIFQEMCLHKPLPRPKQRG---NQLIYVAGG
BLOCKS                             : BBBBBBBBBBB--------------------------------------------------------------------------
FORMULA                            :                                                                              1

CYS_IMPORTANT_RESIDUES             : ----------X----------------------X-C--------------------------C----------C-----------
STRUCTURE_AND_FUNCTION             : B-----------------W------------B----------Y--GGB-B--------------------C-----------B--
NP_036421.2|Human                  : YFRQSLSYLEAYNPSDGTWLRL-ADLQVPRSGLAGCVVGGLLYAVGGRNNSPDGNTDSSALDCYNPMTNQWSPCAPMSVPRNRIG
NP_001103775.1|Mouse               : YFRQSLSYLEAYNPSNGSWLRL-ADLQVPRSGLAGCVVGGLLYAVGGRNNSPDGNTDSSALDCYNPMTNQWSPCASMSVPRNRIG
NP_001094612.1|Cattle              : YFRQSLSYLEAYNPSDGTWLRL-ADLQVPRSGLAGCVVGGLLYAVGGRNNSPDGNTDSSALDCYNPMTNQWSPCASMSVPRNRIG
XP_036702860.1|Blue_whale          : YFRQSLSYLEAYNPSDGTWLRL-ADLQVPRSGLAGCVVGGLLYAVGGRNNSPDGNTDSSALDCYNPMTNQWSPCASMSVPRNRIG
XP_004865558.1|Naked_mole_rat      : YFRQSLSYLEAYNPSDGTWLRL-ADLQVPRSGLAGCVVGGLLYAVGGRNNSPDGNTDSNALDCYNPMTNQWSPCAPMSVPRNRIG
XP_006023504.1|Chinese_alligator   : YYRQSLGFLEAYNPHDGAWLRL-ADLEVPRSGLASCAVGGLLYAVGGRNNSPDGNTDSGALDCYNPMTNQWSPCAPMSVPRNRIG
NP_001008024.1|Western_clawed_frog : YYRQSLSFLEAYNPVDGEWLTL-ASLEMPRSGLAGCVLGGLFYAVGGRNNAPDCNKDSGALDCYNPMNNQWSPCAAMSVPRNRVG
NP_001106948.1|Danio_rerio         : YFRQSLSFLEAFNPCSGAWLRL-ADLQVPRSGLAACVISGLLYAVGGRNNGPDGNMDSHTLDCYNPMNNCWRPCAHMSVPRNRIG
NP_878284.2|Danio_rerio            : YKQHSLDTLEAFDPHKNVWLKL-GSMMSPCSGLGACVLFGLLYTVGGRNLSLQNNTESGSLSCYNPMTNQWTQLAPLNTPRNRVG
XP_002940707.2|Western_clawed_frog : YLQNSLSSMDAFNPQTGEWIKL-ADMLEPRSGLGACIVSGLFYAVGGRNNSCQENTDSDLLSFFNPVTNQWASRAPMNVPRNRVG
BLOCKS                             : ----------------------------------BBBBBBBBBBBBB----------BBBBBBBBBBBBBBBBBBBBBBBB----
FORMULA                            :                       1   2                                            2   3

CYS_IMPORTANT_RESIDUES             : ----------------X-------C----------------------------------------X-----C-------------
STRUCTURE_AND_FUNCTION             : --------Y--GG-----B----Z--------W----------------------Y--GGB------------------W-----
NP_036421.2|Human                  : VGVIDGHIYAVGGSHGCIHHNSVERYEPERDEWHLVAPMLTRRIGVGVAVLNRLLYAVGGFDGTNRLNSAECYYPERNEWRMITA
NP_001103775.1|Mouse               : VGVIDGHIYAVGGSHGCIHHSSVERYEPERDEWHLVAPMLTRRIGVGVAVLNRLLYAVGGFDGTNRLNSAECYYPERNEWRMITP
NP_001094612.1|Cattle              : VGVIDGHIYAVGGSHGCIHHNSVERYEPEGDEWHLVAPMLTRRIGVGVAVLNRLLYAVGGFDGTNRLNSAECYYPERNEWRMITP
XP_036702860.1|Blue_whale          : VGVIDGHIYAVGGSHGCIHHNSVERYEPERDEWHLVAPMLTRRIGVGVAVLNRLLYAVGGFDGTNRLNSAECYYPERNEWRMITP
XP_004865558.1|Naked_mole_rat      : VGVIDGHIYAVGGSHGCIHHNSVERYEPERDEWHLVSPMLTRRIGVGVAVLNRLLYAVGGFDGTNRLNSAECYYPERNEWRLITP
XP_006023504.1|Chinese_alligator   : VGVIDGLIYAVGGSHGCVHHDSVERYDPECDAWERAAPMQTRRIGVGVAVLNRLLYAVGGFDGSARLRSAECYNPERNEWHPIAP
NP_001008024.1|Western_clawed_frog : AGVIDGQIYAVGGSHGCLHHNSVERYDPERDEWHMVSPMKTRRIGVGVAVLNRLLYAVGGFDGTNRLNSAECYYPETDEWKDIAS
NP_001106948.1|Danio_rerio         : VGVIDGMIYAVGGSHGCTHHNSVERYDPERDSWQLVSPMLTRRIGVGVAVINRLLYAVGGFDGTHRLSSAECYNPERDEWRSIAA
NP_878284.2|Danio_rerio            : VGVIDGSIYAVGGSHASTHHNSVERYDPETNRWTFVAPMSVARLGAGVAACGGCLYVVGGFDGDNRWNTVERYQPDTNTWQHVAP
XP_002940707.2|Western_clawed_frog : VAEIDGAIYAAGGSCGSEHHKSVEKYDPDNDRWTFVAPMPTARIGAGVVACRGRLYVVGGFDGDTRWNTVDCYDPEEDQWQPVAS
BLOCKS                             : -------------------------------------------------------------------------------------
FORMULA                            :                                      3   4
```

```
CYS_IMPORTANT_RESIDUES                : ----------C----C-----------------C---------------------------------------------------
STRUCTURE_AND_FUNCTION                : -----------------Y--GGB---------Z--------W----------------------Y--------------GGB---
NP_036421.2|Human                     : MNTIRSGAGVCVLHNCIYAAGGYDGQDQLNSVERYDVETETWTFVAPMKHRRSALGITVHQGRIYVL------------GGYDGH
NP_001103775.1|Mouse                  : MNTIRSGAGVCVLHNCIYAAGGYDGQDQLNSVERYDVETETWTFVAPMRHHRSALGITVHQGKIYVL------------GGYDGH
NP_001094612.1|Cattle                 : MNTIRSGAGVCVLHNCIYAAGGYDGQDQLNSVERYDVETETWTFVAPMKHRRSALGITVHQGRIYVL------------GGYDGH
XP_036702860.1|Blue_whale             : MNTIRSGAGVCVLHNCIYAAGGYDGQDQLNSVERYDVETETWTFVAPMKHRRSALGTTVHQGRIYVL------------GGYDGH
XP_004865558.1|Naked_mole_rat         : MNTIRSGAGVCVLHNCIYAAGGYDGQDQLNSMERYDVEMETWTFVAPMKHRRSALGITVHQGRIYVL------------GGYDGH
XP_006023504.1|Chinese_alligator      : MNTIRSGAGTCALNNCLYAMGGYDGTDQLNSTERYDVEADAWTFVAPMRHRRSALGVTTYQGKIYVL------------GGYDGH
NP_001008024.1|Western_clawed_frog    : MNIVRSGAGACALDTSVYAMGGYDGTDQLNSVERYDVEKDDWTFVAPMRHRRSALGVTVHQGKIYVL------------GGYDGS
NP_001106948.1|Danio_rerio            : MNTVRSGAGVCALGNYIYVMGGYDGTNQLNTVERYDVEKDSWSFSASMRHRRSALGVTTHHGRIYVL------------GGYDGN
NP_878284.2|Danio_rerio               : MNTVRSGLGVVCMDNYLYAVGGYDGQTQLKTMERYNITRDVWEPMASMNHCRSAHGVSVYQCKIFVL------------GGFNQG
XP_002940707.2|Western_clawed_frog    : METIRSGAGVVALDNYLYAVGGYDGTNQLDSVERYNIERNYWEAMAPMKHRRSAHGLTVHQGKIYALGKELKYFLNYNVGGFNAG
BLOCKS                                : -------------------------------------------------------------------------------------
FORMULA                               : 4   5                                          5   6

CYS_IMPORTANT_RESIDUES                : -------C-----------------------------X--------X-X---------------------
STRUCTURE_AND_FUNCTION                : ---------------W------------------------------------------------------
NP_036421.2|Human                     : TFLDSVECYDPDTDTWSEVTRMTSGRSGVGVAVTMEPCRKQIDQQNCTC---------------------
NP_001103775.1|Mouse                  : TFLDSVECYDPDSDTWSEVTRMTSGRSGVGVAVTMEPCRKQIDQQNCTC---------------------
NP_001094612.1|Cattle                 : TFLDSVECYDPDTDTWSEVTRMTSGRSGVGVAVTMEPCRKQIDQQNCTC---------------------
XP_036702860.1|Blue_whale             : TFLDSVECYDPDTDTWSEVTCMTSGRSGVGVAVTMEPCRKQIDQQNCTC---------------------
XP_004865558.1|Naked_mole_rat         : TFLDSVECYDPDTDTWSEVTRMTSGRSGVGVAVTMEPCRKHIDQQNCTC---------------------
XP_006023504.1|Chinese_alligator      : TFLDSVECYDPATDTWTEVTCMTSGRSGVGVAVTMEPCR---DQPGCHC---------------------
NP_001008024.1|Western_clawed_frog    : TFLDGVECYNPATDTWTEVTQMTSGRSGVGVAITMEPCRKQPCGGGLFPERVKDGSHDKKKCCFSSHTKK
NP_001106948.1|Danio_rerio            : TFLDSVECFDPETDSWTEVTHMKSGRSGVGVAVTMEPCHK--ELIPCQC---------------------
NP_878284.2|Danio_rerio               : GFLSSVECYCPASNVWTLVTDMPVGRSGMGVAVTMEPCPGILPEEEEEVDEEM-----------------
XP_002940707.2|Western_clawed_frog    : GFLSSVECYCPDRNEWTEVTVMPTGCSGMSVAVTMEPCPQLCDTNEEN----------------------
BLOCKS                                : ----------------------------------------------------------------------
FORMULA                               : 6
```

Figure S5. Short version of the phylogenetic tree of the KLHL proteins with the Nrf2 binding motif mapped onto it

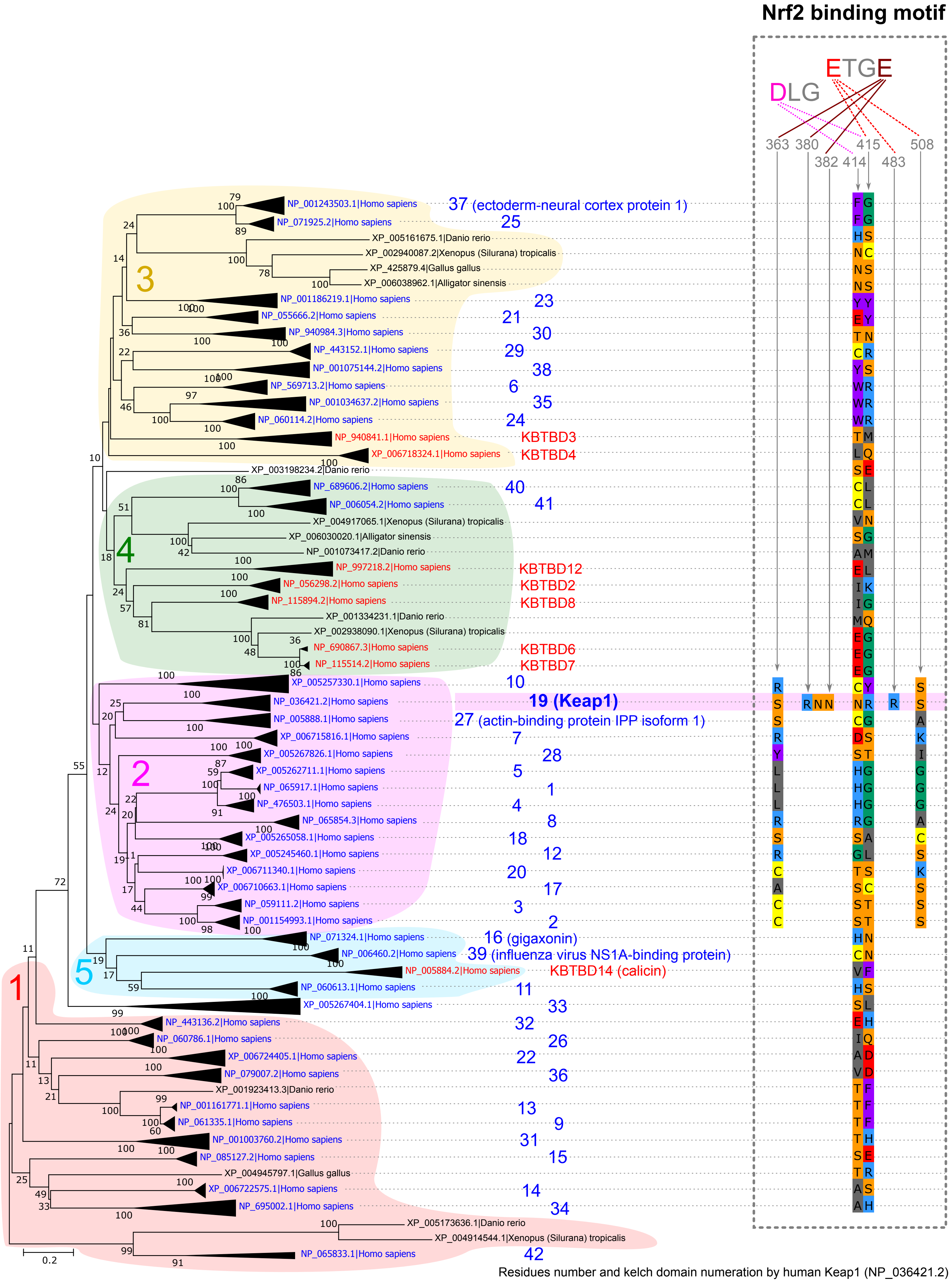

Residues number and kelch domain numeration by human Keap1 (NP_036421.2)

***Figure S6*. Comparison between domain borders produced by the Pfam profile HMM search (top) and those annotated in the Uniprot/SwissProt database (bottom) for human Keap1: (A)** BTB and BACK domains, **(B)** two Kelch domains.

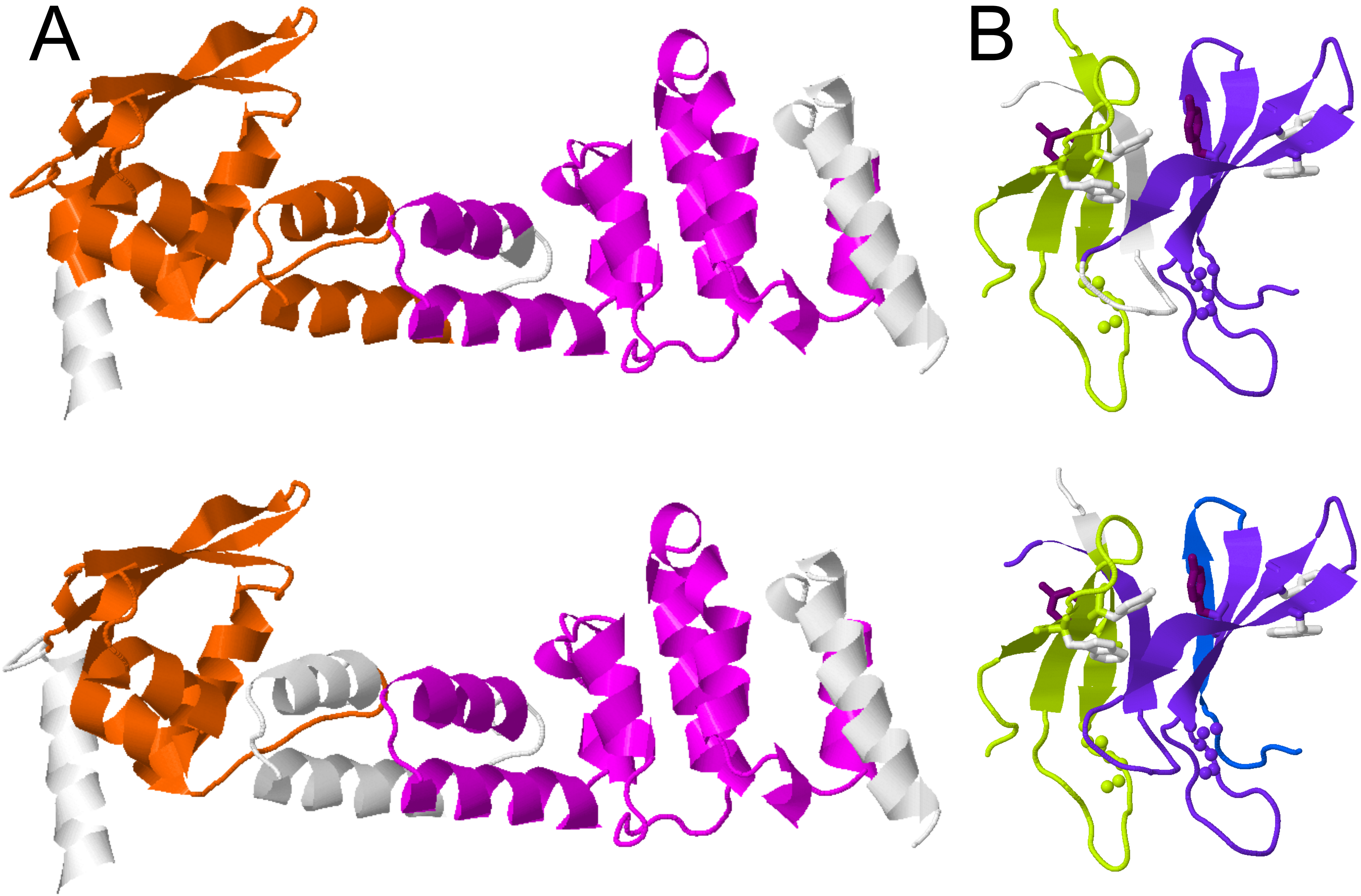

Supplement: Supplementary file 1 [file life-13-01045-s001.zip › Supplementary_file1.pdf]
