# Supplementary material for: Regulation of Cell Proliferation and Nrf2-Mediated Antioxidant Defense: Conservation of Keap1 Cysteines and Nrf2 Binding Site in the Context of the Evolution of KLHL Family
Source: Life (Basel). 2023 Apr 19;13(4):1045. doi: 10.3390/life13041045 (PMC10146909; doi:10.3390/life13041045)
Supplement: Supplementary file 1 [file life-13-01045-s001.zip › Supplementary_file2.pdf]

Supplementary file 2

to the article: "Regulation of Cell Proliferation and Nrf2-Mediated Antioxidant Defense: conservation of Keap1 cysteines and Nrf2 binding site in the context of the evolution of KLHL Family"

Analysis of conservation of several important positions in Keap1. The best known conserved elements of these proteins are the double glycine repeats of Kelch domains (from the presence of which the entire region is sometimes referred to in the literature as DGR, double glycine repeat), present in many proteins from humans to mosquitoes and Drosophila. However, tryptophan, located second from the beginning of the β-strand D (βD) in all six lobes of the Kelch domains, turned out to be an even more conservative amino acid in the entire KLHL protein family. Tryptophan (βD), along with a pair of glycines present at the end of the βB chain and tyrosine residues (βC), regulates the hydrophobic packing between the blades.

In two cases (in Kelch2 (W403) and Kelch3 (W450)) it occurs in all 42 human KLHL proteins (no amino acid repeats this record); in 41 cases in Kelch5 (W544); in 40 - in Kelch4 (W497), however, only 34 times in Kelch1 (W352) and Kelch6 (W591). Trp losses in Kelch1 (W352) are evenly distributed between the KLHL clades: one in clade I, two in clade II, and three in clade 3 and two in clade 4 (See Table S2 below).

The occurrence of glycine within the GG repeats following βB strand is also exceptionally high: 40 out of 42 in Kelch1 (G332 and G333); 41/42 and 40/42 in Kelch2 (G378 and G379); 38/42 and 41/42 in Kelch3 (G429 and G430); 40/42 and 39/42 in Kelch4 (G476 and G477); but then decreases slightly: 39 out of 42 and 38/42 in Kelch5 (G523 and G524, respectively) and especially in Kelch6 - 36/42 and 35/42 (respectively for G570 and G571).

In Kelch1, one G is missing in KLHL42 (clade 1) and in KLHL40 and KLHL41 (clade 4), and in KLHL40 and 41 this motif is generally strongly disturbed. In Kelch2 there is no one G in KLHL11 (clade 4). Kelch3 completely lacks a GG repeat in KLHL21 (clade 3) and lacks one G in KLHL15 and KLHL22 (clade 1) and KLHL41 (clade 4). In Kelch4, the GG repeat is completely absent in the KLHL11 (clade 5) and a single G is missing in the KLHL22 (clade 1) and KLHL30 (clade 3). There is no GG repeat in Kelch5 for KLHL21 (clade 3) and KLHL11 (clade 5) and only single G is observed in KLHL34 (clade 1). In Kelch6 the pair of glycines is missing in KLHL25, KLHL29, KLHL37 and KLHL38 (clade 3), as well as KLHL42 (clade 1), KLHL16 (clade 5) and in KLHL7 and KLHL12 (clade 2).

Table S2. **Detailed description of conservation of cysteines and other functionally and structurally important residues in Keap1 (KLHL19) and other members of the KLHL family**. Asterisk sign * marks residues which have basic residues in their closest neighbors. Chamsa-Chaban cysteine (position 208) which occurs in Keap1 of several long-living species which are tolerant to oxidative stress is marked with double asterisk sign **. Numbering in the first column is given only for the cysteine residues which are present in human Keap1. Ultraconservative positions, i.e. occuring in more than 66% of the sample, are colored yellow. KLHL33 protein which according to our analysis does not belong to any clade specifically is considered for simplicity a member of clade 1 and is marked with the dagger † sign.

**# of C**	**Domain**	**Residue**	**Neighbors of Cys residues / comments for other residues**	**Conserved in human KLHL proteins**	**KLHL family**				
					Clade 1	Clade 2	Clade 3	Clade 4	Clade 5
	**NTR**		**[1–66]**						
1	NTR	C13	A(C13)C	1	--	In Keap1 (KLHL**19**) only	--	--	--
2	NTR	C14*	C(C14)**R**	2	--	**-19**	--	--	--
3	NTR	C23	Q(C23)P	3	--	**-19**, -27	--	--	--
4	NTR	C38*	E(C38)**K**	4	KLHL32	-5, **-19**	KLHL35	--	--
	**BTB**		**[67–178]**						
5	BTB	C77	L/F(C77)D	24	-9, -13, -14, -31, -34, -36	all clade (1-5; -7, -8, -10, -12, -17-20, -27, -28,	--	--	KLHL11; -16;-39
	BTB	D78		41					
	BTB	C95	P/S/**H**(C95)**H**	12	-14	-8	-6, -23-25, -30, -35, -37, 38	-40, -41	--

	BTB	H96		40					
	BTB	K97/R		12/29					
	BTB	L100		39					
	BTB	A101		33					
	BTB	C102	A(C102)G/S/V/C	4	-33†, -34	-8, -39	--	--	--
	BTB	C103	A/S/V(C103)S	18	-14, -26, -31, -32, -36	-2, -3, -10, -12, -17, -20, -39	-23, -24, -29, -37	-40, -41	--
	BTB	S104		38					
	BTB	Y106		39					
	BTB	F107		40					
	BTB	Residues 108-117	Belongs to the α3/ α3.1 structure; important for the Cul3 binding						
	BTB	K108/R	Belongs to the α3/ α3.1 structure; important for the Cul3 binding	6/18					
	BTB	F111		36					
	BTB	E117	φ-xE motif; important for the Cul3 binding	36					
	BTB	Y141	Important for the	40					

			dimerization process and catalytic activity						
6	BTB	C151*	K/D(C151)V	2	-26	-19	--	--	--
	BTB	V152		26					
	BTB	A159		37					
		C160	A(C160)Q	**7**	--	-1, -4, -5, -8, -12, 18	-21	--	--
	BTB	Q163	Important for the dimerization process and catalytic activity	34					
7	BTB	C171	A(C171)/S/C/G/V/X	40	-9, -13-15, -22,-26, -31, -34, -36	all clade (1-5; -7, -8, -10, -12, -17-20, -27, -28, -39)	-6, -21, -23, -24, -29, -30, -35, -37, -38	all clade (-40, -41)	-11, -16,
	BTB	C172	C(C172)E/D/S	16	-15, -22, -36	-1-3, -5, -8, -10, -12, -17, -18, -20, -27, -28	--	--	-16
	BTB	L175		41					
		N183	Belongs to the 3-box residues (181–212) which is important for the catalysis	39					
	**BACK**	[185–285]							
	BACK	C184	N(C184)L/I/V/C/M/A	36	-9, -13, -15, -26, -31, -33†, -34, -42	1-5, -7, -8, -10, -12, -17, -18, -20, -27, -28, -39	-6, -21, -23-25, -29, -30, -37, -38	all clade IV (-40, -41)	-11, -16,

	BACK	L199	Belongs to the 3-box residues (181–212) which is important for the catalysis	34					
8	BACK	C196	G/S/Q/(C196)V/T/X	22	--	-1-5, -7, -10, -12, -17, -18-20, -27, -28	-21, -23, -25, -29, -37, -38	-40, -41	--
	BACK	C208**		4	--	-8, -28	-23	-40	--
	BACK	F211		35					
	BACK	F220		35					
	BACK	L223		37					
9	BACK	C226*	**H**/(C226)Q	2	-32	**-19**	--	--	--
10	BACK	C241*	**R**(C241)E	3	-22, -36	**-19**	--	--	--
	BACK	E242		40					
	BACK	A248		29					
11	BACK	C249	A(C249)I	1	--	**-19**	--	--	--
	BACK	D256		26					
12	BACK	C257	D(C257)E	1	--	**-19**	--	--	--
	BACK	R260		36					
	BACK	R272		36					
13	BACK	C273*	**R(**C273)**H/R**	2	--	**-19**	--	-40	--

	**IVR**	**[286–323]**							
14	IVR	C288*	**K**/A(C288)E	2	-36	**-19**	--	--	--
	IVR	C294	L(C294)P/H/G	4	--	-18	-6, -21, -35	--	--
15	IVR	C297*	**R**/X(C297)**K**/X	24	-9, -13, -14, -26, -31, -36	-1-5; -7, -8, -10, -12, -17-20, -28	-3, -21, -24, -29, -35, -38	--	-11
	IVR	C312	**R**/A(C312)**K**/R/F	4	--	--	-25, 30, 35, 37	--	--
16	IVR	C319*	P(C319)**R**/**K**	2	--	**-19, -**27	--	--	--
	**Kelch1**	**[324–358]**							
	Kelch1	Y329	βB	7					
	Kelch1	G332		40					
	Kelch1	G333		40					
	Kelch1	C338	A/V/**R**(C338)L/**K**	4	-32, -36	-28, -39	--	--	--
	Kelch1	C339	M/S(C339)D/R	3	--	-7	-25, -37	--	--
	Kelch1	E343	βC	16					
	Kelch1	C344	E/D/T(C344)Y/L	8	--	-2, -3, -8, -39	-6, -21, -24, -29	--	--
	Kelch1	Y345	βC	26					
	Kelch1	W352	βD	34					
	Kelch1	S355		15					
	**Kelch2**	**[361–410]**							
	Kelch2	G366	βA	18					
	Kelch2	C366	H/D/T/Q(C366)V/G	6	-14, -15, -26, -	--	--	--	--

					31, -32, -36				
17	Kelch2	C368	V/A/G**(**C368)A/V/T	5	-34	-1, **-19**	-24, -35, -37	--	--
	Kelch2	Y375	βB	30					
	Kelch2	G378		41					
	Kelch2	G379		42					
	Kelch2	E394	βC	12					
18	Kelch2	C395	E/D/W(C395)Y/F/L/W	8	-1, -4, -5	-7, -12, **-19**, -28	-29, -30	--	--
	Kelch2	Y396	βC	28					
	Kelch2	W403	βD	42					
19	Kelch2	C406	P(C406)A	1	--	**-19**	--	--	--
		A407		20					
	**Kelch3**	**[412–456]**							
	Kelch3	R413		40					
	Kelch3	C414	**R**(C414)Y/G/N/R/L	6		-10, -27, -39	-29	-40, -41	--
	Kelch3	G417	βA	19					
	Kelch3	Y426	βB	38					
	Kelch3	G429		41					
	Kelch3	G430		42					
20	Kelch3	C434	G(C434)I	1	--	**-19**	--	--	--
	Kelch3	C436	S/T/R(C436)L/F	4	--	-8, -17, -20	--	40	--

	Kelch3	E441	βC	38					
	Kelch3	Y443	βC	37					
	Kelch3	W450	βD	42					
	**Kelch4**	**[459–504]**							
	Kelch4	G464/ A464	βA	11/19					
	Kelch4	C468	V/G/S/A(C468)		-42	--	-21, -24, -29, -30, -35	--	--
	Kelch4	Y473	βB	34					
	Kelch4	G476		40					
	Kelch4	G477		39					
	Kelch4	C483	G/S/D(C483)L/T	11	--	-1-5, -10, -39	-23, -24	-40, -41	--
	Kelch4	E488	βC	17					
21	Kelch4	C489	E/Q/M/T(C489)Y/F	15	-9, -13, -14, -22, -26, -36	-2, -5, **-19**	-6, -21, -30, -35	--	-11, -16
	Kelch4	Y490	βC	32					
	Kelch4	P492		34					
	Kelch4	W497	βD	41					
	Kelch4	C500	(C500)A	4	--	-1, -4, -5	-38	--	--
	**Kelch5**	**[507–550]**							
	Kelch5	R507		34					

	Kelch5	G511	βA	20					
	Kelch5	C511	**H**/L(C511)M/A/S	4	-9, -13, -31	--	-23	--	
22	Kelch5	C513	3M/V/A(C513)T/S/V/G	5	-9, -13, -36	**-19**	--	--	-16
23	Kelch5	C518	N(C518)I/L	3	--	-8, **-19, -**27	--	--	--
	Kelch5	G523		39					
	Kelch5	G524		38					
	Kelch5	E535	βC	30					
	Kelch5	Y537	βC	40					
	Kelch5	W544	βD	41					
	**Kelch6**	**[553–597]**							
	Kelch6	C557	(C557)G	4	--	--	-6, -23, -24, -35	--	--
	Kelch6	G558	βA	26					
	Kelch6	C559	G/T/Q(C559)A/C/I	5	-14, -26, -42	-7	-38	--	--
	Kelch6	C560	V/I/C(C560)L/A/P/Y	7	-26	-1-5	--	--	-11
	Kelch6	Y567	βB	30					
	Kelch6	G570		36					
	Kelch6	G571		35					
	Kelch6	E582	βC	17					
24	Kelch6	C583	E/L/Q/I(C583)Y/W/F	7	-14, -31	-10, -12, **-19**	-6, -24	--	--

	Kelch6	Y584	βC	30					
	Kelch6	W591	βD	34					
	**Kelch1**	**[598–611]**<br>(loop and finishing β-strand)							
		C602	R(C602)	4	--	-12, -27, -28	--	--	-11
	Kelch1	G605	βA	17					
	Kelch1	C605	A/G/S(C605)V/I	9	-33†	-1, -4, -5	-23, -24, -29, -35	-41	--
	Kelch1	C607	V/I/C/A(C607)V/**H**	5	--	-7, -10, -12	-23	--	-11
	Kelch1	C608	A/S/G/**R**(C608)**T**/V/I/L/C	5	-14, -26, -31, -34	-18	--	--	--
	**CTR**	**[612–624]**							
25	CTR	C613*	P(C613)**R**	1	--	**-19**	--	--	--
26	CTR	C622	N(C622)T	1	--	**-19**	--	--	--
27	CTR	C624	T(C624)	1	--	**-19**	--	--	--
